# Early life and adult stress promote sex dependent changes in hypothalamic miRNAs and environmental enrichment prevents stress-induced miRNA and gene expression changes in rats

**DOI:** 10.1186/s12864-021-08003-4

**Published:** 2021-09-28

**Authors:** Lauren Allen McKibben, Yogesh Dwivedi

**Affiliations:** Present Address: Department of Psychiatry and Behavioral Neurobiology, University of Alabama at Birmingham, SC711 Sparks Center, 1720 2nd Avenue South, Birmingham, AL 35294 USA

**Keywords:** Early life stress, Maternal separation, microRNA, Hypothalamus, Restraint stress, Enrichment, microRNA sequencing, Methylation, Rat

## Abstract

**Background:**

The hypothalamus plays a key role in the stress response. While early life stress (ELS) increases susceptibility to psychiatric disorders including major depressive disorder (MDD), acute stress during adulthood can also precipitate MDD after ELS.

**Aim:**

Here, we tested the expression of miRNAs following ELS and susceptibility to depression-like behavior and whether sex or acute stress exacerbates this response. We also tested whether environmental enrichment (Enr) promotes early life and adult behavioral stress resilience and its effect on hypothalamic miRNA and gene expression. Following rat maternal separation (MS) as an ELS model, Enr from weaning through adulthood, and restraint (RS) as acute adult stress, we tested both animal behavior and miRNA expression in the hypothalamus. Target genes and their enrichment and ontology were analyzed using bioinformatic tools. Target gene expression changes were tested using qPCR, and miRNA promoter methylation was studied using methylated-DNA immunoprecipitation qPCR.

**Results:**

MS, Enr, RS, and sex altered hypothalamic miRNAs, including several previously reported in MS literature: miRs-29, − 124, − 132, − 144, − 504. Sex had a significant effect on the greatest number of miRNAs. Also, Enr reversed downregulation of miR-29b-1-5p and -301b-3p in MS. qPCR showed that MAPK6 and MMP19, targets of miR-301b-3p, were upregulated in MS and reversed by Enr. Additionally, miR-219a was hypermethylated in MS coinciding with decreased miR-219a expression.

**Conclusions:**

This study found that sex plays a critical role in the hypothalamic miRNA response to both ELS and acute stress, with males expressing greater changes following postnatal stress. Moreover, enrichment significantly altered behavior as well as hypothalamic miRNA expression and their gene targets. Because of its role as the initiator of the autonomic stress response and connection to hedonic and motivational behavior, the hypothalamic miRNA landscape may significantly alter both the short and long-term behavioral response to stress.

**Supplementary Information:**

The online version contains supplementary material available at 10.1186/s12864-021-08003-4.

## Background

Early life stress (ELS), namely abuse, neglect, and household dysfunction, is estimated to affect 64% of the US population [1]. This poses a major public health risk as ELS increases susceptibility to psychiatric disorders, including major depressive disorder (MDD). Not only does MDD carry a sizeable economic burden due to lost productivity [2], its symptoms are particularly debilitating. MDD onset has also been strongly associated with a precipitating stressful event, especially within the previous month [3]. Moreover, ELS and stress during adulthood can interact producing more severe or long-lasting symptoms than individually [4], also described as the two/three-hit hypothesis of stress susceptibility [5]. There are also significant sex differences in the response to ELS [6]. In the US, men and women experience similar levels of ELS [7], yet women are twice as likely to develop MDD [8]. There are only a limited number of studies that have examined sex-mediated depressive response to ELS [9].

Previous preclinical and clinical studies have implicated hypothalamic-pituitary-adrenal (HPA)-axis responsiveness in the depressive effects of ELS [10–12]. The HPA axis is the primary neurochemical stress response system whereby corticotropin releasing hormone (CRH) is secreted from the hypothalamus triggering a cascade of hormone release by the pituitary (adrenocorticotropin-releasing hormone-ACTH) and adrenal glands (corticosterone-CORT). Not only is the hypothalamus the initial site of this neuroendocrine response, but it also receives inputs from regions central to emotion processing like the amygdala [13] and frontal cortex [14]. Moreover, it plays a particularly important role in the symptomatology of MDD [15]. The hypothalamus receives sensory cues predicting reward (or lack thereof) and stimulates dopamine downstream [16]. In rodents, disruption of dopaminergic projections to the hypothalamus leads to reduced sucrose preference (i.e. more anhedonia-like behavior) [17]. Similarly, antagonizing hypothalamic GABA receptors increases anxiety-related measures in the elevated plus maze (EPM) such as time spent and number of entries into the closed arms [18] and directly injecting calcium channel inhibitors into the hypothalamus increases swimming behavior in the forced swim test (FST) indicated reduced behavioral despair [19]. These findings point to several neurochemical signaling mechanisms in the hypothalamus which might contribute to increased stress and depression-related behaviors. ELS has also been associated with disturbed HPA function including increased cortisol response to ACTH administration and increased cerebrospinal fluid CRH levels in MDD patients [20, 21]. While our understanding of the HPA stress response and its interaction with ELS has improved over the decades, currently available treatments for MDD are only modestly effective.

Recently, microRNAs (miRNAs) have been proposed as potential therapeutic or biomarker targets for MDD [22–25]. These small non-coding RNAs (~ 22 nucleotides) are synthesized in the cell nucleus as a hairpin loop and then exported into the cytoplasm where the loop is cleaved. This mature, single-stranded miRNA is loaded into the RNA-induced silencing complex (RISC) and targets the 3’UTR of genes with partially matching nucleotide sequences [26]. Canonically, miRNAs block the translation of mRNAs into proteins. Furthermore, because miRNAs are relatively short and can bind to mRNAs without perfect base pairing, they are able to target many different genes concurrently. Only a few studies to date have explored the effect of ELS on miRNAs (reviewed in [27]), one of which employed short duration maternal separation (MS) as a form of enrichment and found increases in miRs-488, − 144, and − 542-5p and decreases in miRs-421 and -376b-5 in hypothalamus [28]. MS has been well established as a rodent model of ELS associated with depression- and anxiety-like behavior [29]. MS protocols vary, but typically rodent pups are separated from their dam for 180 min daily for 12–21 days. Another study combined postnatal MS and restraint stress (RS) during adulthood to test if RS precipitated depressive behavior after MS [30]. Animals who experienced both MS and RS showed increased immobility in the forced swim test and decreased sucrose preference along with increased medial prefrontal cortex expression of miR-124 [30]. Increased miR-124 in the dentate gyrus after 90 min MS has also been reported [31]. While miRNAs serve a primary function to regulate gene expression, they are also regulated by epigenetic modifications such as methylation [32]. Only a few studies have elucidated miRNA methylation changes in psychiatric disorders. In adolescent patients with high risk for MDD, significant hypomethylation was found in miR-4646-3p promoter region [33]. Using chronic CORT administration to induce depression-like behavior in rats, our group found promoter hypomethylation of miR-124-3p [34]. No studies have explored miRNA promoter methylation in an ELS model.

There has been a growing shift toward non-pharmaceutical treatments in mental health. Clinical trials have increasingly pursued behavioral interventions while pharmaceutical-based trials have decreased from 43% in 2007 to 27% by 2018 [35]. Depressed individuals with a history of ELS may respond less to antidepressant drug therapy than patients with no ELS history [36]; however, very little is yet understood about how non-pharmaceutical methods contribute to healthy brain function. Environmental enrichment (Enr) has been found to improve behaviors in rodents related to anxiety [37, 38] and MDD [39] and lower blood CORT levels after RS [38]. Earlier work proposed that Enr reverses HPA dysregulation after ELS [40, 41].

The purpose of the study was: 1) to show whether ELS-induced depressive behavior is associated with miRNA expression changes in the hypothalamus and whether there is an interaction between MS, RS, and sex on miRNA expression; 2) to examine the effect of the interaction between RS, sex and Enr in MS animals on hypothalamic miRNA expression; 3) explore if these MS-induced miRNAs changes in the hypothalamus relate to gene expression changes and whether these miRNAs themselves might be regulated via methylation. To examine these, we assessed the behavioral and physiological consequences of MS and Enr as well as genome-wide changes in miRNA expression using next-generation sequencing. We also used bioinformatic tools to understand the potential functions associated with altered miRNAs and their targets. Finally, we tested the expression of stress-related miRNA gene targets and potential regulation of miRNAs by promoter region methylation.

## Results

### Differential miRNA expression in MS, RS, and enriched animals

**Fig. 1:**
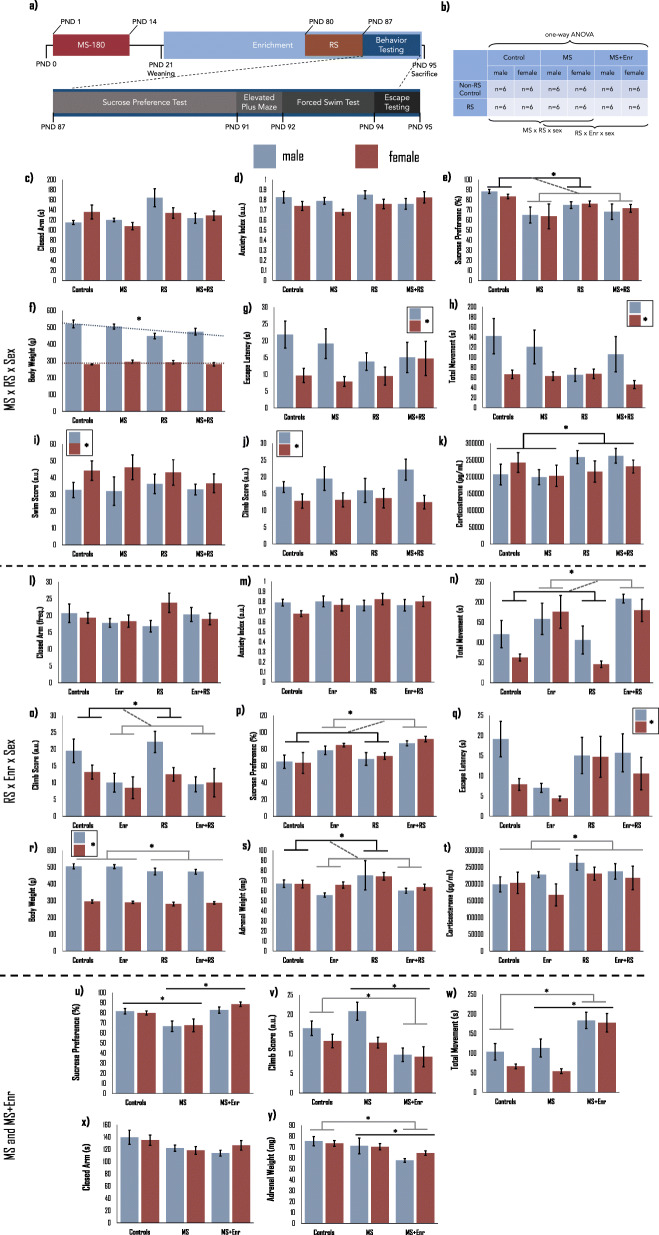
Behavior and physiology associated with maternal separation, restraint stress, sex, and enrichment. A schematic diagram (**a**) shows the timeline of behavioral testing relative to MS, Enr, and RS. MS was conducted for 180 min daily for the first 14 postnatal days. Enr was provided to animals following weaning until tissues were harvested at PND 95. **b)** Statistical comparisons of behavior and miRNA expression were conducted using 3-way ANOVA across MS, RS, and sex (n = 6 animals per group; first 4 columns of Fig. 1b). Another 3-way analysis was conducted across RS, Enr, and sex, only in MS animals (last 4 columns of Fig. 1b). Analysis of Enr as a treatment following MS was conducted using one-way ANOVA with control, MS, and MS + Enr as groups (indicated across the top of Fig. 1b; *n* = 12 animals per group including *n* = 6 RS and n = 6 Non-RS animals per group). **c-k)** Behavioral changes based on the interaction between MS, RS, and sex are shown. Importantly, MS reduced sucrose preference (**e;** F(1,48)=9.24, *p* = 0.004) regardless of RS or sex. Sex was a significant predictor for escape latency (**g;** F(1,48)=7.70, *p* = 0.008), EPM movement (**h;** F(1,48)=9.20, *p* = 0.004), and FST swim and climb scores (**i;** F(1,48)=4.19, *p* = 0.047; **j**;F(1,48)=8.61, *p* = 0.006). **l-t)** In MS animals, a 3-way ANOVA revealed increased EPM movement (**n;** F(1,48)=22.91, *p* < 0.000) and sucrose preference (**p:**(1,48)=16.05, *p* < 0.000) and decreased FST climbing (**o;** F(1,48)=11.92, *p* = 0.001) and adrenal weight (**s;** F(1,48)=5.23, *p* = 0.028) following Enr. RS significantly increased plasma Cort levels (**t;** F(1,48)=4.39, *p* < 0.05).One-way ANOVA comparison of control, MS, and MS + Enr groups (**u-y)** revealed the Enr recovered sucrose preference changes induced by MS (**u;** F(2,69)=12.049, *p* < .001) as well as recovering FST climbing changes in male animals (**v;** F(2, 69)=6.841, *p* < 0.005). Bars represent Mean ± SEM with males in blue and females in red. Dotted linear trend lines in **f** indicate an interaction. Significance: *p < 0.05. Abbreviations: PND-postnatal day, MS-maternal separation, RS-restraint stress, Enr-environmental enrichment

Animals were assigned to MS, RS, and Enr groups or relative controls. As shown in Fig. 1a MS animals were separated for 14 days and enrichment began immediately post-weaning; control animals were handled daily but not separated and were housed in standard conditions. Animals in RS groups were restrained for 7 days prior to behavioral testing and tissue collection; animals assigned to non-RS control groups were briefly handled daily. Finally, RNA isolated from the hypothalamus was sequenced to determine miRNA expression differences across these groups; animal groups for each set of comparisons are shown in Fig. 1b**.**

#### Behavior differences resulting from MS, RS, and sex

We tested for main and interaction effects on physiological and behavioral measures of stress (animals included in this analysis are noted in Fig. 1b by the label “MS x RS x sex”) and detected a significant 3-way interaction between MS, RS and sex on EPM closed arm time (F(1,48)=5.30, *p* = 0.027; Fig. 1c), however there was no significant effect on the EPM index score (*p* > 0.05; Fig. 1d). Follow-up pairwise t-tests showed that RS-only males spent more time in the closed arms compared to MS, MS + RS, and controls (t(22)= − 2.423, *p* = 0.056). There was a significant decrease in sucrose preference for all MS animals compared to all non-MS (F(1,48)=9.24, *p* = 0.004;Fig. 1e). RS significantly altered the effect of sex on body weight (F(1,48)=5.79, *p* = 0.021;Fig. 1f). There was also a significant main effect of sex on escape latency (F(1,48)=7.70, *p* = 0.008;Fig. 1g) with all males exhibiting higher escape latency than females. We found significantly decreased total movement in the EPM in all female animals compared to males (F(1,48)=9.20, *p* = 0.004;Fig. 1h). We also found that forced swim test (FST) swim scores were higher in female than male animals (F(1,48)=4.19, *p* = 0.047;Fig. 1i**)** but climb scores were higher in males than females (F(1,48)=8.61, *p* = 0.006;Fig. 1j); there were no differences in FST float score.

#### The interaction of MS, RS, and sex on corticosterone levels

We compared plasma CORT level across groups using an independent samples t-tests (Fig. 1k) but there were no significant differences (*p* > 0.05). In males, RS significantly increased CORT level compared to controls and MS-only males (t(22)= − 2.511, *p* < .05).

#### The effect of estrus on behavior and miRNAs

Because of the significant sex differences in behavior and concerns for the effect of female sex hormones on stress-related changes, we tested female animals for estrus phase via vaginal lavage. Representative microscope images of cell cytology and group behavior differences are shown in Supplementary Fig. 1. Because there were no significant behavior differences between the estrus phase groups further analyses did not include this variable. Detailed results are described in Supplementary Table 5.

#### Behavior differences resulting from RS, enrichment, and sex in MS rats

A RS x Enr x Sex ANOVA was conducted to identify the effect of these factors and their interaction on behavior. For this analysis, only MS animals were considered because we were primarily interested in exploring enrichment as a preventative measure as opposed to its use simply as enrichment in normal controls (animals included in this analysis are noted in Fig. 1b by the label “RS x Enr x sex”).

There were no significant effects of MS, RS, or Enr on EPM closed arm frequency (Fig. 1l) or the anxiety index (Fig. 1m**)**. Enr significantly increased total movement in the EPM, regardless of RS (F(1,48)=22.91, *p* < 0.000;Fig. 1n). All Enr animals regardless of RS showed a significant reduction in climb scores compared to non-enriched animals (F(1,48)=11.92, *p* = 0.001;Fig. 1o). Enrichment significantly increased sucrose preference in MS animals regardless of whether they had received RS (F(1,48)=16.05, *p* < 0.000;Fig. 1p). There were no significant effects on escape latency (*p* > 0.05;Fig. 1q**)**. As expected, there was a significant main effect of sex on body weight (F(1,48)=495.06, *p* < 0.000), but we also found that RS decreased body weight (F(1,48)=4.82, *p* = 0.034;Fig. 1r) compared to all animals without RS. Adrenal weight was significantly reduced by Enr regardless of RS (F(1,48)=5.23, *p* = 0.028;Fig. 1s). Finally, we found that RS increased plasma CORT levels (Fig. 1t; F(1,48)=4.39, *p* < 0.05).

#### miRNAs altered by the interaction of MS, RS, and sex

3-way ANOVA was used to detect significant miRNA expression differences associated with MS, RS, and sex (Table 1). The fold change column headers indicate the two comparison groups and direction of change (e.g. a fold change value of 1.33 for “MS:control” indicates 1.33-times fold change increase in expression in MS relative to control animals). Specific *p* values are listed alongside fold change in Table 1. Only miR-26a-3p was significantly affected by the 3-way interaction (*p* < 0.05). 5 miRNAs were affected by the interaction of MS and RS, 11 by sex and MS, and 46 by sex and RS (*p* < 0.05). Alone, sex significantly altered 24 miRNAs while MS affected 28 and RS affected 43 (*p* < 0.05). The miRNAs with the greatest fold change were miR-547-3p which was 2.45-fold downregulated in all females compared to all males (*p* < 0.05), miR-144-3p which was 2.3-fold downregulated in MS males compared to male controls (*p* < 0.05), and miR-23a-5p which was increased 1.97-fold in all females as compared to males (*p* < 0.05).

**Table 1:** miRNAs altered by the interaction between maternal separation, restraint stress, and sex

		*CPM-Fold Change*					
Accession ID	miRNA	MS: Control				*p*	*Sig.*
*maternal separation*							
*MIMAT0012855*	*miR-666-5p*	1.33				*0.046*	*
*MIMAT0017819*	*miR-3557-5p*	1.28				*0.044*	*
*MIMAT0000838*	*miR-132-3p*	1.28				*0.005*	*
*MIMAT0003379*	*miR-378a-3p*	1.28				*0.024*	*
*MIMAT0017158*	*miR-212-5p*	1.25				*0.008*	*
*MIMAT0003378*	*miR-378a-5p*	1.21				*0.026*	*
*MIMAT0035730*	*miR-1843b-5p*	1.20				*0.025*	*
*MIMAT0000859*	*miR-181b-5p*	1.19				*0.020*	*
*MIMAT0003205*	*miR-409a-3p*	1.18				*0.030*	*
*MIMAT0000550*	*miR-323-3p*	1.16				*0.024*	*
*MIMAT0001626*	*miR-431*	1.12				*0.033*	*
*MIMAT0003194*	*miR-376c-3p*	1.10				*0.050*	*
*MIMAT0000832*	*miR-126a-3p*	1.09				*0.028*	*
*MIMAT0004706*	*let-7e-3p*	1.08				*0.028*	*
*MIMAT0004732*	*miR-135a-3p*	−1.11				*0.046*	*
*MIMAT0000805*	*miR-30e-5p*	− 1.18				*0.034*	*
*MIMAT0004726*	*miR-101a-5p*	− 1.20				*0.042*	*
*MIMAT0017026*	*miR-301a-5p*	−1.20				*0.046*	*
*MIMAT0017143*	*miR-186-3p*	−1.21				*0.022*	*
*MIMAT0005328*	*miR-673-5p*	−1.21				*0.006*	*
*MIMAT0035720*	*let-7 g-3p*	−1.23				*0.031*	*
*MIMAT0017839*	*miR-3065-5p*	−1.24				*0.033*	*
*MIMAT0000812*	*miR-33-5p*	−1.31				*0.039*	*
*MIMAT0017230*	*miR-497-3p*	−1.39				*0.024*	*
*MIMAT0005304*	*miR-301b-3p*	−1.56				*0.011*	*
*MIMAT0005445*	*miR-29b-1-5p*	−1.71				*0.012*	*
*MIMAT0017154*	*miR-206-5p*	−1.77				*0.017*	*
*MIMAT0000850*	*miR-144-3p*	−1.78				*0.011*	*
*restraint stress*		RS:Control					
*MIMAT0017881*	*miR-3586-3p*	1.26				*0.016*	*
*MIMAT0000791*	*miR-22-3p*	1.21				*0.014*	*
*MIMAT0000784*	*miR-15b-5p*	1.20				*0.008*	*
*MIMAT0003380*	*miR-505-3p*	1.20				*0.011*	*
*MIMAT0003193*	*miR-494-3p*	1.20				*0.029*	*
*MIMAT0017840*	*miR-3065-3p*	1.19				*0.038*	*
*MIMAT0003175*	*miR-543-3p*	1.17				*0.038*	*
*MIMAT0000794*	*miR-24-3p*	1.17				*0.012*	*
*MIMAT0000792*	*miR-23a-3p*	1.17				*0.034*	*
*MIMAT0000793*	*miR-23b-3p*	1.17				*0.002*	**
*MIMAT0004714*	*miR-26b-3p*	1.17				*0.030*	*
*MIMAT0035719*	*let-7 g-5p*	1.13				*0.040*	*
*MIMAT0000840*	*miR-134-5p*	−1.09				*0.040*	*
*MIMAT0017307*	*miR-434-5p*	−1.10				*0.047*	*
*MIMAT0017306*	*miR-425-3p*	−1.10				*0.016*	*
*MIMAT0005339*	*miR-873-5p*	−1.11				*0.037*	*
*MIMAT0017031*	*miR-329-5p*	−1.11				*0.010*	*
*MIMAT0017137*	*miR-181c-3p*	−1.13				*0.040*	*
*MIMAT0000902*	*miR-300-3p*	−1.13				*0.023*	*
*MIMAT0005332*	*miR-708-3p*	−1.14				*0.043*	*
*MIMAT0017117*	*miR-127-5p*	−1.14				*0.009*	*
*MIMAT0004655*	*miR-345-3p*	−1.14				*0.015*	*
*MIMAT0005341*	*miR-488-3p*	−1.15				*0.026*	*
*MIMAT0003192*	*miR-379-5p*	−1.15				*0.001*	***
*MIMAT0024847*	*miR-1843a-5p*	−1.15				*0.030*	*
*MIMAT0003199*	*miR-381-3p*	− 1.16				*0.012*	*
*MIMAT0000781*	*miR-9a-5p*	−1.17				*0.016*	*
*MIMAT0004728*	*miR-124-5p*	−1.18				*0.027*	*
*MIMAT0000802*	*miR-29a-3p*	−1.18				*0.012*	*
*MIMAT0004740*	*miR-218a-2-3p*	−1.19				*0.012*	*
*MIMAT0005312*	*miR-411-5p*	−1.20				*0.002*	**
*MIMAT0017334*	*miR-146b-3p*	− 1.20				*0.010*	*
*MIMAT0000833*	*miR-127-3p*	−1.21				*0.014*	*
*MIMAT0005308*	*miR-380-5p*	−1.21				*0.005*	**
*MIMAT0017360*	*miR-582-3p*	−1.22				*0.016*	*
*MIMAT0017828*	*miR-3559-3p*	−1.22				*0.018*	*
*MIMAT0017291*	*miR-879-3p*	−1.22				*0.007*	*
*MIMAT0000587*	*miR-341*	−1.23				*0.004*	**
*MIMAT0005325*	*miR-598-3p*	−1.24				*0.002*	**
*MIMAT0001534*	*miR-448-3p*	−1.27				*0.024*	*
*MIMAT0000814*	*miR-34c-5p*	−1.28				*0.014*	*
*MIMAT0024856*	*miR-6216*	−1.39				*0.038*	*
*MIMAT0017896*	*miR-3593-5p*	−1.84				*0.045*	*
*sex*		Female:Male					
*MIMAT0004712*	*miR-23a-5p*	1.97				*0.031*	*
*MIMAT0004739*	*miR-204-3p*	1.58				*0.027*	*
*MIMAT0000877*	*miR-204-5p*	1.29				*0.023*	*
*MIMAT0017802*	*miR-3547*	1.27				*0.039*	*
*MIMAT0004729*	*miR-125a-3p*	1.21				*0.045*	*
*MIMAT0003378*	*miR-378a-5p*	1.20				*0.030*	*
*MIMAT0001549*	*miR-365-3p*	1.19				*0.016*	*
*MIMAT0017175*	*miR-421-3p*	1.18				*0.000*	***
*MIMAT0004648*	*miR-339-3p*	1.16				*0.043*	*
*MIMAT0004740*	*miR-218a-2-3p*	1.15				*0.041*	*
*MIMAT0017306*	*miR-425-3p*	1.11				*0.011*	*
*MIMAT0000774*	*let-7a-5p*	1.11				*0.043*	*
*MIMAT0000796*	*miR-26a-5p*	1.09				*0.006*	*
*MIMAT0005335*	*miR-758-3p*	−1.11				*0.034*	*
*MIMAT0005299*	*miR-181d-5p*	−1.12				*0.046*	*
*MIMAT0003207*	*miR-369-3p*	−1.13				*0.022*	*
*MIMAT0017867*	*miR-3579*	−1.20				*0.039*	*
*MIMAT0017192*	*miR-433-5p*	−1.22				*0.038*	*
*MIMAT0005311*	*miR-410-3p*	−1.27				*0.007*	*
*MIMAT0017136*	*miR-154-3p*	−1.37				*0.022*	*
*MIMAT0017130*	*miR-144-5p*	−1.49				*0.017*	*
*MIMAT0001633*	*miR-451-5p*	−1.73				*0.006*	*
*MIMAT0000850*	*miR-144-3p*	−1.83				*0.008*	*
*MIMAT0012851*	*miR-547-3p*	−2.45				*0.000*	***
*MS x RS*		MS: Control		MS + RS: RS			
*MIMAT0017117*	*miR-127-5p*	−1.12		1.11		*0.028*	*
*MIMAT0005325*	*miR-598-3p*	−1.15		1.14		*0.038*	*
*MIMAT0017851*	*miR-3571*	−1.31		1.42		*0.020*	*
*MIMAT0017818*	*miR-3556b*	−1.31		1.01		*0.043*	*
*MIMAT0001534*	*miR-448-3p*	−1.37		1.13		*0.031*	*
*MS x sex*		MS Male: Control Male		MS Female: Control Female			
*MIMAT0012830*	*miR-504*	1.28		−1.20		*0.044*	*
*MIMAT0005278*	*miR-466b-5p*	1.03		−1.19		*0.047*	*
*MIMAT0000839, MIMAT0003126*	*miR-133a/b-3p*	−1.12		1.49		*0.047*	*
*MIMAT0037263*	*miR-1b*	−1.19		1.59		*0.043*	*
*MIMAT0017131*	*miR-145-3p*	−1.29		1.83		*0.049*	*
*MIMAT0017838*	*miR-218b*	−1.33		1.10		*0.023*	*
*MIMAT0017851*	*miR-3571*	−1.35		1.43		*0.014*	*
*MIMAT0017823*	*miR-3596d*	−1.39		1.08		*0.042*	*
*MIMAT0017886*	*miR-3596a*	−1.45		1.11		*0.041*	*
*MIMAT0017887*	*miR-3588*	−1.58		1.25		*0.030*	*
*MIMAT0000850*	*miR-144-3p*	−2.30		−1.16		*0.037*	*
*RS x sex*		RS Male: Control Male		RS Female: Control Female			
*MIMAT0000886*	*miR-216a-5p*	1.48		−1.35		*0.035*	*
*MIMAT0017136*	*miR-154-3p*	1.46		−1.15		*0.044*	*
*MIMAT0000801*	*miR-29b-3p*	1.43		−1.09		*0.040*	*
*MIMAT0003195*	*miR-376b-5p*	1.41		−1.07		*0.040*	*
*MIMAT0012833*	*miR-582-5p*	1.39		−1.27		*0.025*	*
*MIMAT0004743*	*miR-300-5p*	1.38		−1.16		*0.007*	*
*MIMAT0000552*	*miR-301a-3p*	1.37		−1.15		*0.049*	*
*MIMAT0012834*	*miR-592*	1.35		−1.18		*0.033*	*
*MIMAT0000577*	*miR-337-3p*	1.32		−1.12		*0.050*	*
*MIMAT0005301*	*miR-188-5p*	1.30		−1.11		*0.032*	*
*MIMAT0004791*	*miR-379-3p*	1.30		−1.16		*0.042*	*
*MIMAT0003196*	*miR-376b-3p*	1.27		− 1.16		*0.014*	*
*MIMAT0017143*	*miR-186-3p*	1.24		−1.14		*0.031*	*
*MIMAT0024845*	*miR-3068-5p*	1.23		−1.18		*0.022*	*
*MIMAT0017223*	*miR-374-3p*	1.20		−1.22		*0.046*	*
*MIMAT0017146*	*miR-191a-3p*	1.19		−1.11		*0.027*	*
*MIMAT0000813*	*miR-34b-5p*	1.18		−1.46		*0.016*	*
*MIMAT0017111*	*miR-98-3p*	1.17		−1.17		*0.038*	*
*MIMAT0000888*	*miR-218a-5p*	1.15		−1.22		*0.017*	*
*MIMAT0017213*	*miR-541-3p*	1.14		−1.24		*0.007*	*
*MIMAT0017127*	*miR-138-2-3p*	1.14		−1.12		*0.018*	*
*MIMAT0017100*	*miR-26a-3p*	1.13		−1.08		*0.024*	*
*MIMAT0005335*	*miR-758-3p*	1.11		−1.11		*0.041*	*
*MIMAT0003191*	*miR-493-3p*	1.09		−1.17		*0.022*	*
*MIMAT0017212*	*miR-539-3p*	1.08		−1.30		*0.034*	*
*MIMAT0024846*	*miR-3068-3p*	1.07		−1.13		*0.012*	*
*MIMAT0017874*	*miR-3583-3p*	1.07		−1.17		*0.048*	*
*MIMAT0017307*	*miR-434-5p*	1.03		−1.26		*0.011*	*
*MIMAT0000791*	*miR-22-3p*	1.02		1.41		*0.028*	*
*MIMAT0004733*	*miR-136-3p*	1.02		−1.52		*0.048*	*
*MIMAT0005339*	*miR-873-5p*	1.00		−1.24		*0.037*	*
*MIMAT0017137*	*miR-181c-3p*	−1.00		−1.26		*0.041*	*
*MIMAT0017360*	*miR-582-3p*	−1.02		−1.46		*0.033*	*
*MIMAT0017117*	*miR-127-5p*	−1.03		− 1.27		*0.036*	*
*MIMAT0001534*	*miR-448-3p*	−1.03		− 1.54		*0.047*	*
*MIMAT0005331*	*miR-708-5p*	− 1.04		1.17		*0.033*	*
*MIMAT0005308*	*miR-380-5p*	−1.06		− 1.41		*0.041*	*
*MIMAT0004705*	*let-7b-3p*	−1.06		1.27		*0.041*	*
*MIMAT0004648*	*miR-339-3p*	−1.15		1.18		*0.043*	*
*MIMAT0017871*	*miR-3596b*	−1.19		1.48		*0.012*	*
*MIMAT0017886*	*miR-3596a*	−1.20		1.37		*0.031*	*
*MIMAT0017877*	*miR-3596c*	−1.25		1.36		*0.024*	*
*MIMAT0000903*	*miR-320-3p*	−1.28		1.47		*0.037*	*
*MIMAT0017094*	*miR-16-3p*	−1.28		1.18		*0.017*	*
*MIMAT0005313*	*miR-423-3p*	−1.30		1.52		*0.039*	*
*MIMAT0017866*	*miR-3578*	−1.33		1.35		*0.031*	*
*MS x RS x sex*		MS Male: C Male	MS Female: C Female	MS RS Male: RS Male	MS RS Female: RS Female		
*MIMAT0017100*	*miR-26a-3p*	1.21	−1.02	− 1.07	1.25	*0.009*	*

All miRNAs with a significant main effect or interaction effect are listed here. CPM-fold change is presented as a ratio one group’s miRNA expression over another group (usually the comparison control). Positive values indicate upregulation and negative values show downregulation. *p < 0.05, **p < 0.005, ***p < 0.001. Abbreviations: CPM-counts per million, MS-maternal separation, RS-restraint stress, C-control

#### miRNAs altered by the interaction of RS, enrichment, and sex in maternally separated animals

miRNAs significantly altered by RS, Enr, sex or their interaction (based on 3-way ANOVA) in MS animals are reported in Table 2 along with their fold change and specific significance (*p* value). The 3-way interaction (RS, Enr, and sex) had a significant effect on 32 miRNAs. The largest number of miRNAs were affected by the interaction between Enr and sex, with 56 miRNAs significantly altered. There was a significant interaction between RS and Enr on miR-135b-3p expression (*p* < 0.05). miR-666-3p was significantly affected by the interaction between RS and sex (*p* < 0.05). We detected 2 miRNAs associated with RS main effect: miR-338-5p and − 341 (*p* < 0.05). There was a main effect of Enr on 39 miRNAs and sex affected expression of 14 miRNAs. The greatest fold change in expression was found for miR-547-3p with females showing a 2.51-fold downregulation compared to males (*p* < 0.05) regardless of RS or Enr. Next, miR-539-5p showed the next greatest fold change at 1.84-fold increased expression in Enr females compared to non-Enr females (*p* < 0.05).

**Table 2:** miRNAs altered by the interaction between restraint stress, environmental enrichment, and sex in maternally separated rats

		*CPM-Fold Change*					
Accession ID	miRNA					*p*	*Sig.*
*restraint stress*		RS:Control					
*MIMAT0004646*	*miR-338-5p*	1.20				*0.018*	*
*MIMAT0000587*	*miR-341*	−1.13				*0.043*	*
*enrichment*		Enr:Non-Enr					
*MIMAT0017895*	*miR-3592*	1.37				*0.002*	**
*MIMAT0017094*	*miR-16-3p*	1.30				*0.002*	**
*MIMAT0017130*	*miR-144-5p*	1.28				*0.036*	*
*MIMAT0017158*	*miR-212-5p*	1.28				*0.022*	*
*MIMAT0017818*	*miR-3556b*	1.27				*0.001*	***
*MIMAT0017836*	*miR-9b-3p*	1.26				*0.022*	*
*MIMAT0005299*	*miR-181d-5p*	1.26				*0.002*	**
*MIMAT0000857*	*miR-181c-5p*	1.25				*0.007*	*
*MIMAT0017357*	*miR-362-3p*	1.24				*0.007*	*
*MIMAT0000820*	*miR-99a-5p*	1.23				*0.024*	*
*MIMAT0017850*	*miR-3570*	1.22				*0.015*	*
*MIMAT0017823*	*miR-3596d*	1.22				*0.010*	*
*MIMAT0005341*	*miR-488-3p*	1.20				*0.021*	*
*MIMAT0017360*	*miR-582-3p*	1.20				*0.029*	*
*MIMAT0017867*	*miR-3579*	1.18				*0.028*	*
*MIMAT0003207*	*miR-369-3p*	1.17				*0.005*	*
*MIMAT0005282*	*miR-872-5p*	1.14				*0.003*	**
*MIMAT0000867*	*miR-192-5p*	1.14				*0.027*	*
*MIMAT0004710*	*miR-17-1-3p*	1.13				*0.031*	*
*MIMAT0017031*	*miR-329-5p*	1.13				*0.009*	*
*MIMAT0003123*	*miR-377-3p*	1.13				*0.009*	*
*MIMAT0003204*	*miR-409a-5p*	1.12				*0.047*	*
*MIMAT0000553*	*miR-324-5p*	−1.09				*0.049*	*
*MIMAT0017371*	*miR-666-3p*	−1.13				*0.012*	*
*MIMAT0000794*	*miR-24-3p*	−1.14				*0.022*	*
*MIMAT0000562*	*let-7d-5p*	−1.16				*0.022*	*
*MIMAT0000784*	*miR-15b-5p*	−1.16				*0.020*	*
*MIMAT0035726*	*miR-149-5p*	−1.17				*0.029*	*
*MIMAT0004729*	*miR-125a-3p*	− 1.18				*0.035*	*
*MIMAT0017874*	*miR-3583-3p*	−1.21				*0.000*	***
*MIMAT0012845*	*miR-935*	−1.21				*0.028*	*
*MIMAT0017318*	*miR-873-3p*	−1.22				*0.031*	*
*MIMAT0000570*	*miR-331-3p*	−1.23				*0.022*	*
*MIMAT0000853*	*miR-150-5p*	−1.26				*0.033*	*
*MIMAT0001549*	*miR-365-3p*	−1.27				*0.004*	**
*MIMAT0001543*	*miR-449a-5p*	−1.33				*0.035*	*
*MIMAT0004742*	*miR-296-3p*	−1.36				*0.027*	*
*MIMAT0035752*	*miR-762*	−1.37				*0.029*	*
*MIMAT0000839, MIMAT0003126*	*miR-133a/b-3p*	− 1.42				*0.026*	*
*sex*		Female:Male					
*MIMAT0035719*	*let-7 g-5p*	1.15				*0.030*	*
*MIMAT0000819*	*miR-98-5p*	1.14				*0.008*	*
*MIMAT0003154*	*miR-29c-5p*	1.13				*0.033*	*
*MIMAT0017175*	*miR-421-3p*	1.12				*0.002*	**
*MIMAT0005309*	*miR-384-5p*	1.11				*0.022*	*
*MIMAT0000796*	*miR-26a-5p*	1.09				*0.012*	*
*MIMAT0024846*	*miR-3068-3p*	−1.07				*0.023*	*
*MIMAT0005342*	*miR-652-3p*	−1.10				*0.015*	*
*MIMAT0005278*	*miR-466b-5p*	−1.10				*0.035*	*
*MIMAT0005311*	*miR-410-3p*	−1.16				*0.050*	*
*MIMAT0004715*	*miR-27a-5p*	−1.18				*0.027*	*
*MIMAT0000887*	*miR-217-5p*	−1.23				*0.039*	*
*MIMAT0025049*	*miR-344i*	−1.25				*0.008*	*
*MIMAT0012851*	*miR-547-3p*	−2.51				*0.000*	***
*RS x Enr*		Enr:Non-Enr		Enr + RS:Non-Enr + RS			
*MIMAT0017043*	*miR-135b-3p*	−1.04		1.27		*0.022*	*
*RS x sex*		RS Male:Control Male		RS Female:Control Female			
*MIMAT0017371*	*miR-666-3p*	1.12		−1.09		*0.043*	*
*Enr x sex*		Enr Male:Non-Enr Male		Enr Female:Non-Enr Female			
*MIMAT0017887*	*miR-3588*	1.45		−1.11		*0.035*	*
*MIMAT0004705*	*let-7b-3p*	1.23		−1.09		*0.014*	*
*MIMAT0017877*	*miR-3596c*	1.23		−1.12		*0.049*	*
*MIMAT0000574*	*miR-140-3p*	1.22		−1.18		*0.036*	*
*MIMAT0000583*	*miR-339-5p*	1.20		−1.17		*0.016*	*
*MIMAT0003378*	*miR-378a-5p*	1.16		−1.36		*0.009*	*
*MIMAT0003213*	*miR-503-5p*	1.15		−1.47		*0.033*	*
*MIMAT0017109*	*miR-93-3p*	1.13		−1.20		*0.048*	*
*MIMAT0000830*	*miR-125b-5p*	1.11		−1.21		*0.028*	*
*MIMAT0004648*	*miR-339-3p*	1.10		− 1.27		*0.036*	*
*MIMAT0000608*	*miR-351-5p*	1.10		−1.71		*0.049*	*
*MIMAT0005299*	*miR-181d-5p*	1.09		1.46		*0.047*	*
*MIMAT0000792*	*miR-23a-3p*	1.08		−1.24		*0.045*	*
*MIMAT0004739*	*miR-204-3p*	1.07		−1.75		*0.034*	*
*MIMAT0017224*	*miR-503-3p*	1.07		−1.54		*0.048*	*
*MIMAT0025067*	*miR-6328*	1.06		−1.34		*0.043*	*
*MIMAT0005282*	*miR-872-5p*	1.04		1.26		*0.030*	*
*MIMAT0000774*	*let-7a-5p*	1.01		−1.16		*0.031*	*
*MIMAT0001549*	*miR-365-3p*	−1.07		−1.48		*0.038*	*
*MIMAT0005335*	*miR-758-3p*	−1.07		1.22		*0.017*	*
*MIMAT0005311*	*miR-410-3p*	−1.08		1.36		*0.016*	*
*MIMAT0025070*	*miR-6331*	−1.11		1.36		*0.012*	*
*MIMAT0004718*	*miR-29a-5p*	−1.11		1.28		*0.038*	*
*MIMAT0005287*	*miR-879-5p*	−1.14		1.28		*0.048*	*
*MIMAT0017136*	*miR-154-3p*	− 1.15		1.70		*0.024*	*
*MIMAT0017102*	*miR-31a-3p*	−1.21		1.32		*0.040*	*
*MIMAT0001628*	*miR-433-3p*	−1.22		1.24		*0.003*	**
*MIMAT0017111*	*miR-98-3p*	−1.23		1.13		*0.030*	*
*MIMAT0000568*	*miR-330-3p*	−1.23		1.15		*0.046*	*
*MIMAT0003381*	*miR-499-5p*	−1.24		1.18		*0.042*	*
*MIMAT0000611*	*miR-135b-5p*	−1.25		1.30		*0.020*	*
*MIMAT0005279*	*miR-466c-5p*	−1.25		1.41		*0.020*	*
*MIMAT0004791*	*miR-379-3p*	−1.27		1.32		*0.034*	*
*MIMAT0000813*	*miR-34b-5p*	−1.27		1.38		*0.014*	*
*MIMAT0000557*	*miR-325-5p*	−1.28		1.35		*0.017*	*
*MIMAT0017211*	*miR-540-5p*	−1.29		1.28		*0.011*	*
*MIMAT0000613*	*miR-151-5p*	−1.30		1.25		*0.043*	*
*MIMAT0000811*	*miR-32-5p*	−1.31		1.28		*0.038*	*
*MIMAT0000865*	*miR-190a-5p*	−1.31		1.20		*0.026*	*
*MIMAT0017104*	*miR-33-3p*	−1.32		1.16		*0.041*	*
*MIMAT0012833*	*miR-582-5p*	−1.32		1.47		*0.038*	*
*MIMAT0012834*	*miR-592*	−1.33		1.38		*0.022*	*
*MIMAT0000577*	*miR-337-3p*	−1.33		1.29		*0.041*	*
*MIMAT0017302*	*miR-380-3p*	−1.34		1.32		*0.034*	*
*MIMAT0024845*	*miR-3068-5p*	−1.34		1.12		*0.037*	*
*MIMAT0000787*	*miR-18a-5p*	−1.35		1.28		*0.023*	*
*MIMAT0017192*	*miR-433-5p*	−1.39		1.27		*0.011*	*
*MIMAT0000789*	*miR-19a-3p*	−1.42		1.36		*0.044*	*
*MIMAT0003195*	*miR-376b-5p*	−1.43		1.21		*0.048*	*
*MIMAT0000552*	*miR-301a-3p*	−1.44		1.30		*0.028*	*
*MIMAT0000825*	*miR-106b-5p*	−1.45		1.23		*0.049*	*
*MIMAT0000801*	*miR-29b-3p*	−1.46		1.35		*0.039*	*
*MIMAT0000855*	*miR-153-3p*	−1.49		1.39		*0.027*	*
*MIMAT0000842*	*miR-136-5p*	−1.51		1.41		*0.021*	*
*MIMAT0017135*	*miR-153-5p*	−1.55		1.35		*0.022*	*
*MIMAT0003176*	*miR-539-5p*	−1.59		1.84		*0.020*	*
*RS x Enr x sex*		Enr Male: Non-Enr Male	Enr Female: Non-Enr Female	Enr + RS Male: Non-Enr RS Male	Enr RS Female: Non-Enr RS Female		
*MIMAT0005322*	*miR-532-5p*	1.32	−1.03	−1.04	1.24	*0.041*	*
*MIMAT0017090*	*let-7f-2-3p*	1.27	1.02	−1.28	1.67	*0.024*	*
*MIMAT0000827*	*miR-122-5p*	1.21	−1.39	− 1.04	1.17	*0.038*	*
*MIMAT0017139*	*miR-181b-1-3p*	1.20	−1.18	−1.13	1.25	*0.013*	*
*MIMAT0035748*	*miR-452-5p*	1.18	−1.30	− 1.11	1.40	*0.024*	*
*MIMAT0000803*	*miR-29c-3p*	1.17	1.01	−1.14	1.37	*0.048*	*
*MIMAT0004705*	*let-7b-3p*	1.16	1.13	1.31	−1.33	*0.025*	*
*MIMAT0000615*	*miR-101b-3p*	1.12	−1.09	−1.15	1.31	*0.040*	*
*MIMAT0012838*	*miR-653-5p*	1.12	−1.11	− 1.12	1.49	*0.026*	*
*MIMAT0012831*	*miR-544-3p*	1.12	−1.12	−1.34	1.47	*0.024*	*
*MIMAT0017143*	*miR-186-3p*	1.09	−1.08	− 1.47	1.26	*0.027*	*
*MIMAT0024846*	*miR-3068-3p*	1.09	−1.03	−1.08	1.12	*0.010*	*
*MIMAT0005282*	*miR-872-5p*	1.08	1.10	−1.00	1.44	*0.041*	*
*MIMAT0004711*	*miR-21-3p*	1.06	−1.41	− 1.38	1.24	*0.026*	*
*MIMAT0017877*	*miR-3596c*	1.06	1.12	1.43	−1.40	*0.020*	*
*MIMAT0017033*	*miR-331-5p*	1.05	−1.10	−1.04	1.18	*0.013*	*
*MIMAT0017871*	*miR-3596b*	−1.01	1.15	1.34	−1.42	*0.010*	*
*MIMAT0000791*	*miR-22-3p*	−1.04	1.11	1.15	−1.35	*0.023*	*
*MIMAT0000774*	*let-7a-5p*	−1.07	−1.06	1.10	−1.26	*0.029*	*
*MIMAT0000862*	*miR-185-5p*	−1.12	−1.01	1.19	−1.31	*0.019*	*
*MIMAT0000560*	*miR-326-3p*	−1.14	1.04	1.13	−1.32	*0.037*	*
*MIMAT0000810*	*miR-31a-5p*	−1.16	1.05	1.26	−1.28	*0.036*	*
*MIMAT0017802*	*miR-3547*	−1.18	−1.01	1.31	−1.59	*0.027*	*
*MIMAT0000562*	*let-7d-5p*	−1.23	−1.08	1.08	−1.44	*0.027*	*
*MIMAT0025067*	*miR-6328*	−1.23	−1.19	1.44	−1.50	*0.027*	*
*MIMAT0035726*	*miR-149-5p*	−1.23	−1.09	1.07	−1.43	*0.045*	*
*MIMAT0000554*	*miR-324-3p*	−1.24	−1.09	1.33	−1.52	*0.023*	*
*MIMAT0004641*	*miR-330-5p*	−1.24	−1.00	1.07	−1.20	*0.022*	*
*MIMAT0012844*	*miR-665*	−1.30	−1.06	1.11	−1.36	*0.039*	*
*MIMAT0012845*	*miR-935*	−1.33	−1.18	1.18	−1.59	*0.035*	*
*MIMAT0000853*	*miR-150-5p*	−1.48	−1.08	1.15	−1.71	*0.018*	*
*MIMAT0017885*	*miR-702-3p*	−1.54	−1.11	1.24	−1.68	*0.039*	*

All animal subjects in this comparison received MS-180. miRNAs with a significant main effect or interaction effect are listed here. CPM-fold change is presented as a ratio one group’s miRNA expression over another group (usually the comparison control). Positive values indicate upregulation and negative values show downregulation. *p < 0.05, **p < 0.005, ***p < 0.001. Abbreviations: CPM-counts per million, MS-maternal separation, RS-restraint stress, Enr-environmental enrichment, C-control

### miRNA-mediated hypothalamic signaling changes after MS and enrichment

#### Enrichment as prevention of depression- and anxiety-related behavior after MS

Our primary focus for this study was to elucidate hypothalamic miRNA changes resulting from MS and environmental enrichment. One-way ANOVA was used to compare controls, MS, and MS + Enr animals. Each group (controls, MS, and MS + Enr) consisted of 24 animals (12 male and 12 female) including RS animals; animals included in this analysis are noted in Fig. 1b as “one-way ANOVA”. There were significant group differences in sucrose preference (F(2,69)=12.049, *p* < .001); MS significantly decreased sucrose preference (*p* < 0.005*)* which was returned to normal levels by Enr (*p* < 0.001; Fig. 1u). Climb score (Fig. 1v**)** in the FST was significantly decreased by Enr as compared to both control (*p <* 0.05*)* and MS (*p* < 0.005) animals (F(2,69)=6.841, *p* < 0.005). In the EPM, Enr significantly increased total movement (Fig. 1w) compared to controls (*p* < 0.001) and MS (p < 0.001) animals (F(2,69)=17.077, *p* < 0.001). One-way ANOVA revealed significant differences (F(2,69)=3.445, *p* < 0.05) in EPM closed arm time (Fig. 1x), but follow-up pairwise t-tests only showed a nonsignificant reduction in MS (*p* = 0.077) and MS + Enr (*p* = 0.08) groups compared to controls. Enrichment significantly reduced adrenal weight (Fig. 1y) compared to both control (*p* < 0.005) and MS (*p <* 0.05) groups (F(2,69)=6.275, *p* < 0.005). Plasma CORT levels showed no significant group differences (F(2,69)=0.485, *p* > 0.05).

#### miRNA expression changes after MS and MS + Enrichment

A one-way ANOVA comparing control, MS, and MS + Enr miRNA counts per million (CPM) expression values showed that 29 miRNAs were significantly upregulated by MS and 21 were significantly downregulated. These changes include miRNAs which were only affected by MS as well as those only affected by MS + Enr or by both MS and MS + Enr. CPM-fold change and *p* values are shown in Table 3 and describe specific expression changes across each of the three groups. miRs-144-3p and − 206-5p exhibited the greatest fold change at 1.783- and 1.766-fold downregulation in MS animals compared to controls (*p* < 0.05). miR-29b-1-5p, −301b-3p, and 3065-5p showed a treatment response to Enr in that MS significantly downregulated their expression (*p* < 0.05) and Enr, at least partially, reversed this effect. Changes specific to male and female animals are shown in Supplementary Tables 1 and 2.

**Table 3:** miRNAs significantly altered by MS and Enrichment

			CPM-Fold Change			*p (one-way ANOVA)*	
Accession ID	Chromosomal Location	miRNA	MS:C	MS + Enr:C	MS + Enr:MS		*sig.*
*upregulated by MS*							
*MIMAT0017819*	chr18: 56726128–56,726,240	*miR-3557-5p*	1.282	1.359	1.060	*0.043*	*b,d,*
*MIMAT0000838*	chr10: 62014995–62,015,095	*miR-132-3p*	1.278	1.394	1.091	*0.000*	*a,b,d,f*
*MIMAT0003379*	chr18: 56726150–56,726,214	*miR-378a-3p*	1.276	1.362	1.068	*0.019*	*a,b,d,*
*MIMAT0026467*	chr11: 16097346–16,097,433	*miR-125b-2-3p*	1.252	1.425	1.138	*0.034*	*b,d,f*
*MIMAT0017158*	chr10: 62014702–62,014,812	*miR-212-5p*	1.252	1.598	1.276	*0.000*	*a,b,c,d,f*
*MIMAT0035730*	chr13: 77065446–77,065,507	*miR-1843b-5p*	1.201	1.263	1.051	*0.015*	*a,b,d,*
*MIMAT0000859*	chr13: 54952903–54,953,012	*miR-181b-5p*	1.195	1.373	1.149	*0.004*	*a,b,d,f*
*MIMAT0003205*	chr6: 133893419–133,893,495	*miR-409a-3p*	1.176	1.324	1.125	*0.000*	*a,b,d,f*
*MIMAT0000822*	chr8: 45746948–45,747,027	*miR-100-5p*	1.176	1.436	1.221	*0.002*	*b,d,f*
*MIMAT0000550*	chr6: 133861199–133,861,284	*miR-323-3p*	1.163	1.275	1.097	*0.001*	*a,b,d,f*
*MIMAT0000820*	chr11: 16052153–16,052,233	*miR-99a-5p*	1.161	1.431	1.232	*0.000*	*b,c,d,f*
*MIMAT0017163*	chrX: 3684480–3,684,588	*miR-221-5p*	1.130	1.305	1.156	*0.001*	*b,c,d,f*
*MIMAT0017217*	chr6: 133699509–133,699,592	*miR-493-5p*	1.124	1.220	1.085	*0.046*	*b,d,f*
*MIMAT0003202*	chr6: 133884178–133,884,257	*miR-382-3p*	1.123	1.310	1.167	*0.002*	*b,c,d,f*
*MIMAT0001626*	chr6: 133711425–133,711,538	*miR-431*	1.118	1.225	1.096	*0.002*	*a,b,d,f*
*MIMAT0000858*	chr3: 23150352–23,150,468	*miR-181a-5p*	1.117	1.343	1.203	*0.021*	*b,d,f*
*MIMAT0017123*	chr10: 62014995–62,015,095	*miR-132-5p*	1.115	1.249	1.120	*0.002*	*a,b,d,f*
*MIMAT0005299*	chr19: 25290051–25,290,133	*miR-181d-5p*	1.113	1.399	1.257	*0.000*	*b,c,d,f*
*MIMAT0003177*	chr6: 133892655–133,892,744	*miR-541-5p*	1.108	1.265	1.142	*0.012*	*b,d,f*
*MIMAT0005314*	chr8: 117354821–117,354,903	*miR-425-5p*	1.105	1.239	1.121	*0.023*	*b,d,f*
*MIMAT0003194*	chr6: 133872439–133,872,522	*miR-376c-3p*	1.103	1.185	1.074	*0.002*	*a,b,d,f*
*MIMAT0024843*	chr1: 143360224–143,360,287	*miR-1839-5p*	1.103	1.237	1.122	*0.014*	*b,d,f*
*MIMAT0017870*	chr6: 133893418–133,893,497	*miR-409b*	1.101	1.173	1.065	*0.045*	*b,d,f*
*MIMAT0003204*	chr6: 133893419–133,893,495	*miR-409a-5p*	1.086	1.220	1.123	*0.003*	*b,c,d,f*
*MIMAT0004706*	chr1: 59704381–59,704,473	*let-7e-3p*	1.082	1.087	1.005	*0.037*	*a,b,d,*
*MIMAT0004710*	chr15: 100179879–100,179,962	*miR-17-1-3p*	1.069	1.212	1.134	*0.001*	*b,c,d,f*
*MIMAT0003200*	chr6: 133877124–133,877,205	*miR-487b-3p*	1.069	1.183	1.106	*0.043*	*b,d,f*
*MIMAT0003207*	chr6: 133893693–133,893,771	*miR-369-3p*	1.061	1.237	1.166	*0.000*	*b,c,d,f*
*MIMAT0005282*	chr5: 113657727–113,657,807	*miR-872-5p*	1.046	1.193	1.141	*0.001*	*b,c,d,f*
*downregulated by MS*							
*MIMAT0000850*	chr10: 65291365–65,291,447	*miR-144-3p*	−1.783	−1.701	1.048	*0.024*	*a,d,*
*MIMAT0017154*	chr9: 26791764–26,791,847	*miR-206-5p*	−1.766	−1.707	1.035	*0.008*	*a,b,d,*
*MIMAT0005445*	chr4: 58344310–58,344,390	*miR-29b-1-5p*	−1.708	− 1.520	1.124	*0.012*	*a,b,d,e,*
*MIMAT0005304*	chr11: 88129426–88,129,503	*miR-301b-3p*	−1.564	− 1.335	1.171	*0.036*	*a,d,e,*
*MIMAT0017230*	chr10: 56844977–56,845,045	*miR-497-3p*	−1.386	−1.582	− 1.142	*0.001*	*a,b,d,f*
*MIMAT0000889*	chr20: 3816158–3,816,267	*miR-219a-5p*	−1.314	−1.504	− 1.144	*0.010*	*b,d,f*
*MIMAT0000812*	chr7: 123431612–123,431,680	*miR-33-5p*	−1.311	−1.620	− 1.235	*0.003*	*a,b,d,f*
*MIMAT0001543*	chr2: 44897601–44,897,691	*miR-449a-5p*	−1.251	−1.660	− 1.327	*0.001*	*b,c,d,f*
*MIMAT0017839*	chr10: 109195234–109,195,336	*miR-3065-5p*	−1.238	−1.139	1.087	*0.045*	*a,d,*
*MIMAT0035720*	chr8: 114874457–114,874,544	*let-7 g-3p*	−1.231	−1.288	− 1.046	*0.017*	*a,b,d,*
*MIMAT0005328*	chr6: 133691381–133,691,463	*miR-673-5p*	−1.210	−1.306	− 1.080	*0.000*	*a,b,d,f*
*MIMAT0017143*	chr2: 263873759–263,873,844	*miR-186-3p*	−1.205	−1.253	− 1.040	*0.015*	*a,b,d,*
*MIMAT0017026*	chr10: 74417746–74,417,845	*miR-301a-5p*	−1.203	−1.341	− 1.115	*0.008*	*a,b,d,f*
*MIMAT0000805*	chr5: 139702872–139,702,963	*miR-30e-5p*	−1.178	−1.238	− 1.052	*0.007*	*a,b,d,f*
*MIMAT0004707*	chr7: 66802731–66,802,815	*let-7i-3p*	−1.168	−1.326	− 1.135	*0.006*	*b,d,f*
*MIMAT0000581*	*chr10: 109195251–109,195,316*	*miR-338-3p*	−1.147	−1.277	− 1.113	*0.024*	*b,d,f*
*MIMAT0017093*	chr2: 165605923–165,606,020	*miR-15b-3p*	−1.144	−1.174	− 1.027	*0.042*	*b,d,*
*MIMAT0000587*	chr6: 133733240–133,733,335	*miR-341*	−1.131	−1.196	− 1.057	*0.020*	*b,d,f*
*MIMAT0004732*	chr7: 32894877–32,894,976	*miR-135a-3p*	−1.114	−1.189	− 1.068	*0.007*	*b,d,f*
*MIMAT0000815*	chr5: 167092491–167,092,592	*miR-34a-5p*	−1.090	−1.241	− 1.139	*0.036*	*b,d,f*
*MIMAT0000570*	chr7: 34881095–34,881,190	*miR-331-3p*	−1.067	−1.315	− 1.233	*0.001*	*b,c,d,f*

^a^ controls vs. MS

^b^ controls vs. MS + Enr

^c^ MS vs. MS + Enr

^d^ controls vs. MS and MS + Enr

^e^ controls and MS + Enr vs. MS (recovered by Enr)

^f^ controls and MS vs. MS + Enr

Significant changes in miRNA expression after MS and Enr based on one-way ANOVA are shown. Listed miRNAs are split by those upregulated vs. downregulated by MS. CPM-fold change value was calculated as a ratio between two group’s CPM expression. Positive values indicate increased expression and negative values show reduced expression in the numerator group. The full one-way ANOVA significance is listed as *p*. Specific group differences are listed as sig. Abbreviations: CPM-counts per million, MS-maternal separation, Enr-environmental enrichment, C-control

**Fig. 2:**
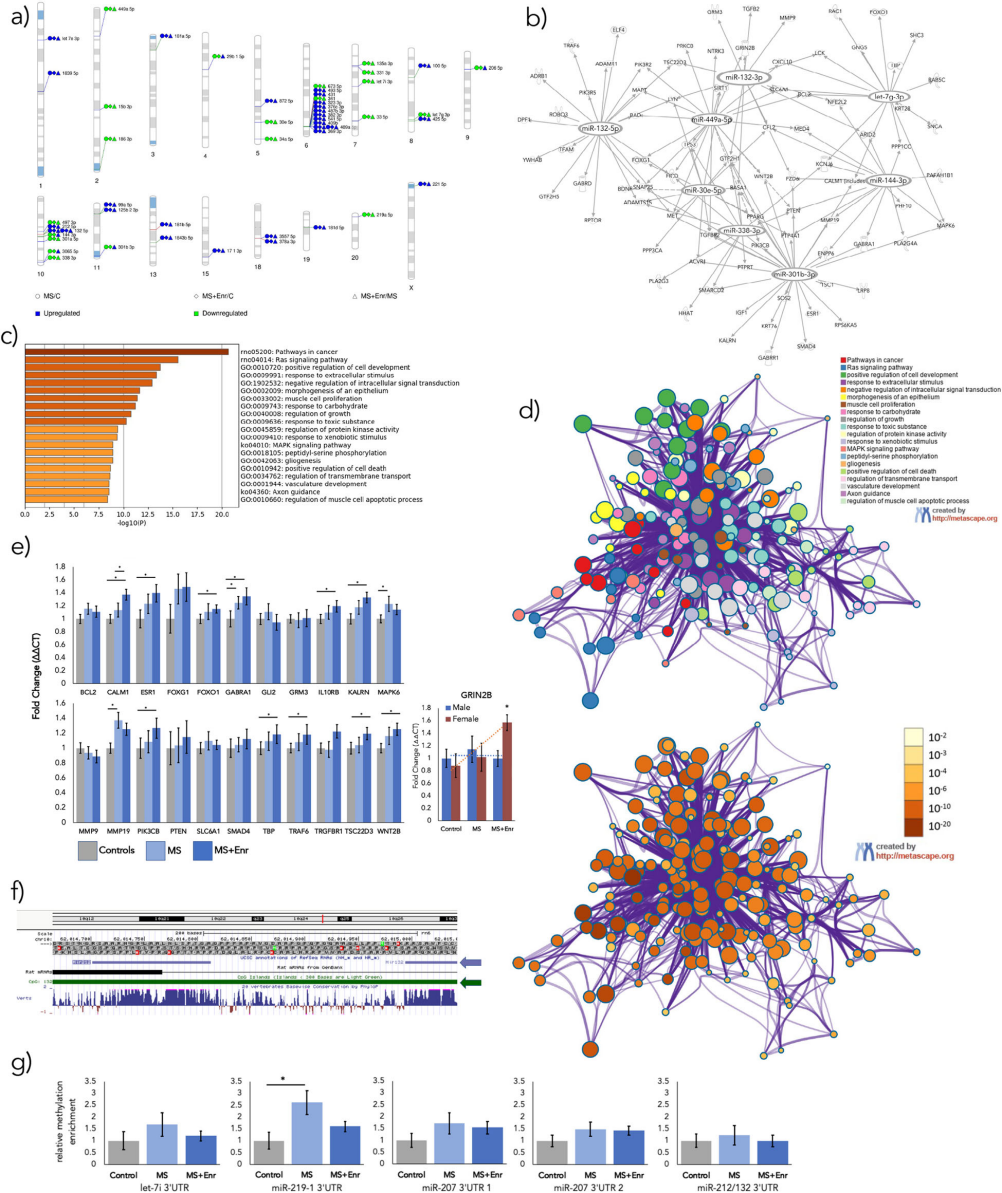
miRNA, gene targets, and ontologies associated with maternal separation and enrichment. a two sizeable groups of miRNAs altered by MS and Enr localized onto chromosomes 6 and 10. Direction of fold change in MS relative to controls, MS + Enr relative to controls, and MS + Enr relatve to MS are represented as circles, diamonds, and triangles, respectively (blue indicates upregulation and green, downregulation). **b** miRNA gene target network shows that 8 significantly altered miRNAs target many stress-related genes and share many targets. **c** MetascapeGO revealed functions most associated with significant miRNAs’ gene targets (Top 3: rno05200-“pathways in cancer”, rno04014-“Ras signaling pathway”, and GO:0010720-“postive regulation of cell development”; more significant bars are darker colored). **d** Metascape also mapped similar ontologies based on common member genes (top map) and significance (bottom map). There is a high level of overlap in membership of these gene targets in Metascape ontologies**. e** Changes in miRNA gene target expression was tested using qPCR. Bars represent M ± SEM and significance were determined by independent samples t-test (*n* = 24 animals per group) and one-way ANOVA (for GRIN2B). GABRA1, MAPK6, and MMP19 were significantly upregulated by MS (t(46)> 2.035, *p* < 0.048) and MAPK6 and MMP19 were partially recovered by Enr. GRIN2B was the only gene affected by the interaction between sex and group (F(2, 72)=3.703, *p* = 0.030). **f** CPG islands were identified near miR-promoter region using the UCSC genome browser; the blue arrow shows miR-212 to the left and miR-132 on the right. The row marked in green shows a CPG island. **g** methylation in the 3’UTR of miR-219-1 was significantly increased by MS (t(30)=2.29, *p* = 0.029); due to limited tissue availability, methylation was tested in *n* = 8 animals per group (compared to *n* = 12 for gene and miRNA expression)**.** For miR-207, methylation in two distinct regions of one CPG island was tested. Significance: *p < 0.05. *Abbreviations: MS-maternal separation, C-controls, Enr-environmental enrichment, UTR-untranslated region, SEM-standard error of the mean*

We visualized the chromosomal location of each significantly altered miRNA in Fig. 2a**.** 12 miRNAs grouped together on chromosome 6 and each of these were upregulated by MS except for miRs-673-5p and − 341. 7 miRNAs grouped on chromosome 10.

#### miRNA gene targets

We used Ingenuity Pathway Analysis (IPA; Hilden, Germany) to determine which miRNAs associated with MS and Enr shared common stress and depression-related gene targets and had the greatest number of these targets. Thus, miRNA-gene target maps were created based on significantly altered miRNAs and their gene targets. To determine hub miRNAs (i.e. those with the most targets and the most shared common targets), we filtered out miRNAs with 10 or fewer gene connections. 3418 gene targets were identified. miRs-132-5p, − 132-3p, −449a-5p, 30e-5p, − 338-3p, −301b-3p, − 144-3p, and let-7 g-3p were hub miRNAs forming a highly integrated network with many shared gene targets (Fig. 2b). Gene ontology (GO) analysis (Fig. 2c) confirmed the involvement of these miRNA gene targets in MAPK signaling as well as Ras signaling, ion binding, and neuron part ontologies. The top ontologies for GO analysis are further described in Supplementary File 1. Hierarchical clustering of the GO terms in Metascape revealed that these pathways and functions were highly overlapped (Fig. 2d). Stress-related gene targets were selected for expression based on a literature search for anxiety, depression, stress, and related molecular pathways (Supplementary Table 3); we also included those genes with the greatest number of targeting miRNAs. 2-way ANOVA revealed that only GRIN2B expression was significantly altered (F(2,72)=3.703, *p* = 0.030) by the interaction between sex and group (control, MS, and MS + Enr) with only females showing increased expression following Enr, though not in controls or MS animals (Fig. 2e**)**. Using pairwise t-tests, we found significant upregulation of GABRA1 (t(46)=2.035, *p* = 0.048), MAPK6 (t(46)=2.307, *p* = 0.026), and MMP19 (t(46)=2.156, *p* = 0.036) in MS (*n* = 24) animals compared to controls (n = 24) (Fig. 2e). Several genes were upregulated in MS + Enr (n = 24) animals compared to controls (n = 24), including CALM1 (t(46)=4.307, *p* < 0.000), ESR1 (t(46)=2.520, *p* = 0.015), FOXO1 (t(46)=2.137, *p* = 0.038), GABRA1 (t(46)=2.384, *p* = 0.021), IL10RB (t(46)=2.271, *p* = 0.028), KALRN (t(46)=3.634, *p* = 0.001), PIK3CB (t(46)=3.077, *p* = 0.004), TBP (t(46)=2.307, *p* = 0.026), TRAF6 (t(46)=2.320, *p* = 0.025), TSC22D3 (t(46)=2.313, *p* = 0.025), and WNT2B (t(46)=2.697, *p* = 0.010). Only two genes showed expression changes following MS that returned to the level of controls following Enr: MAPK6 and MMP19.

#### Exploring methylation as a miRNA regulatory mechanism

Of the 50 miRNAs significantly altered by MS, we found 5 miRNAs (miRs-219a, − 207, − 132, − 212, and let-7i) with CPG islands within 1000 kb of their promoter region. miRs-132 and -212 localized closely on chromosome 10 and shared a CPG island in their promoter regions (Fig. 2f). Pairwise t-test revealed increased methylation at the promoter region of miR-219a in MS (2.61-fold increase, t(30)=2.29, *p* = 0.029; *n* = 4 male and n = 4 female per group) but not MS + Enr animals (Fig. 2g). There were no significant group differences in methylation for let-7i, miR-207, or miR-132/212 (*p* > 0.05).

## Discussion

In this study, we present the first genome-wide profiling of miRNAs in the hypothalamus following MS and RS. We also parsed sex and estrus phase differences in miRNA expression resulting from these stress paradigms. Finally, we show miRNA changes underlying the use of environmental enrichment as a preventative for ELS-induced depressive-behavior. We found that 29 miRNAs were upregulated and 21 were downregulated by MS. Out of 21 downregulated miRNAs, 3 miRNAs (miR-29b-1-5p, −301b-3p, and − 3065-5p) showed expression levels similar to controls in MS + Enr animals. Chromosomal localization revealed two large groups of MS-induced miRNAs on chromosome 6 and chromosome 10. Based on significantly altered miRNAs in MS, we detected over 3418 miRNA gene targets. qPCR-based expression analysis showed that GABRA1, MAPK6, and MMP19 were significantly increased in MS animals compared to controls. Interestingly, MAPK6 and MMP19 expression were reversed by Enr in MS animals. miRNA-gene target mapping revealed several miRNA regulatory hubs including miR-301b-3p, − 132-3p, − 132-5p, −449a-5p, −30e-5p, − 338-3p, − 144-3p, and let-7 g-3p. GO analysis confirmed that these miRNA gene targets were significantly involved in MAPK signaling. Finally, we found that a CPG island near the promoter of miR-219 was hypermethylated in MS but not significantly reversed by Enr.

MDD onset is usually preceded by a recent stressful event [3]. We did not find that RS precipitated a depression-like phenotype in MS animals. In female MS animals, RS increased escape latency but still this did not reach levels associated with learned helplessness (> 20 s latency [42];). Alternatively, each group variable (MS and RS) was associated with unique behavioral profiles. MS significantly decreased sucrose preference indicating increased anhedonia whereas RS decreased total movement in the EPM. However, RS-only males spent increased time in the closed arms of the EPM which has been mostly associated with increased anxiety [43]. RS animals also exhibited increased CORT levels compared to controls and MS without RS. Studies on ELS often report altered stress reactivity meaning that CORT may be more elevated following an acute stressor in individuals who experience ELS compared to those who have no ELS history [44]. Our data potentially support this, but it also shows that RS was sufficient to increase CORT levels whereas MS was not. The majority of behavioral differences were across sex. Males showed increased escape latency, increased anxiety index, increased EPM total movement, and increased climbing in the FST compared to females. Females showed increased swimming in the FST. There have been mixed reports regarding behavioral profiles following MS [45, 46], but sex differences are well documented [47], especially in the FST [48].

Only one miRNA, miR-26a-3p was significantly associated with the interaction between MS, RS, and sex. In this interaction, MS males showed increased expression of miR-26a compared to control males, while MS did not affect its expression in females. However, in RS animals, MS only increased miR-26a expression in females. miR-26a targets HTR1A, one of the serotonin receptors and is upregulated by antidepressants, fluoxetine and reboxetine [49]; however, this was only tested in males. A few other studies have shown significant changes in miR-26 following stress including increased expression in mouse prefrontal cortex [50] as well as in male rats prefrontal cortex who experienced repeated and ancestral stress [51]. Sex differences following ELS, especially stress sensitivity in males prior to puberty, may result from interactions between stress neurobiology and peripubertal sex hormone changes [52]. Future studies should systematically elucidate sex differences in how timing of stress affect miR-26, among others, and its gene targets such as HTR1A. miR-3593 showed the greatest fold change in RS vs. non-RS animals. Cattaneo, Cattane [53] found that hippocampal miR-3593 was downregulated in a rodent prenatal ELS model, but there are no reports in RS or other acute stress. In MS animals, we tested for an interaction between RS, Enr, and sex. In contrast to the 3-way interaction between MS, RS, and sex, there were several miRNAs significantly affected by the 3-way interaction between RS, Enr, and sex in MS animals. Interestingly, Enr in RS animals caused the opposite fold change direction as compared to Enr in non-RS animals. For example, Enr alone was associated with a 1.54-fold *decrease* in miR-702-3p expression compared to controls; Enr + RS males showed a 1.24-fold *increase* in miR-702-3p. This consistently occurred for almost every miRNA. This finding suggests that Enr and RS exhibit opposing miRNA profiles across many miRNAs.

Two large groups of miRNAs associated with MS were colocalized on chromosome 6 and 10. Several significant miRNAs within close proximity could indicate regulation by a common mechanism such as competitive endogenous RNAs [54]. Additionally, when miRNA are closely colocalized they may be transcribed in tandem as a cluster which may indicate similar or dependent functions as well as common regulatory elements [55]. Of the tested gene targets, CALM1 is most closely localized near (~ 9500 kilobases) the miRNA cluster on chromosome 6. Another gene, Delta-like noncanonical notch ligand 1 (DLK1), was identified extremely close (~ 100 kilobases) to the chromosome 6 cluster. DLK1 interacts with NOTCH signaling pathways and is especially involved in pituitary gland development [56]. Furthermore, after adolescence, DLK1 is expressed solely in neuroendocrine tissues in adults. DLK1 and NOTCH signaling changes as mediated by miRNAs may an important avenue for future stress and depression-related studies.

There are previous reports which suggest that estrus phase significantly alters the behavioral response to stress [57]. Our findings showed that only the FST was affected by estrus timing. Proestrus/diestrus (p/d) animals showed decreased swimming, yet increased climbing compared to estrus/metestrus (e/m) animals. This could be attributed to the increased ratio of MS to control animals in p/d group; however, no other behaviors showed significant differences. Kokras, Antoniou [48] also found that animals in the proestrus and diestrus phase exhibited greater climbing duration following treatment with sertraline compared to animals in estrus. A total of 6 miRNAs showed differential expression between the p/d and e/m groups. Of these, miRs-362-3p, − 374-5p, and − 384-5p are localized on the X chromosome. MiR-384-5p has been shown to regulate SOX9, a member of the NFkB pathway [58]. SOX 9 is also an embryonic transcription factor which responds to estrogen during sex determination [59]. Our findings, in general, do not support a particularly robust contribution of estrus timing to stress susceptibility.

In order to identify miRNAs and their regulatory pathways specific to MS and Enr, we compared miRNA expression between control (non-MS) animals, MS animals without Enr, and MS + Enr. While there were a greater number of upregulated miRNAs in MS compared to controls, downregulated miRNAs exhibited greater fold change differences between MS and controls. miRs-144-3p, − 206-5p, −29b-1-5p, and -301b-3p showed the greatest decrease in expression. In an augmented maternal care model using MS, Vogel Ciernia, Laufer [28] reported increased hypothalamic miR-144 expression. Since this model produces increased maternal care behaviors, this could be considered a form of enrichment. However, in comparison, our MS + Enr animals showed similar expression of miR-144 as the MS group. Several miR-29 family miRNAs have been reported in both rodent and human ELS studies. Uchida, Hara [30] found an increase in miR-29a following MS. On the other hand, Cattane, Mora [60] found increased miR-29b-3p and -29c-3p in healthy individuals with a history of ELS. In humans, at least, resilient individuals show opposite expression changes compared to our MS animals. Moreover, our findings show that Enr dampened the effect of MS on miR-29b-1-5p expression. miRNA gene target network mapping revealed several miRNA hubs for potential gene regulation. In another study of 180-min MS, miR-132 was upregulated compared to control [30]. We found similar increases in expression in both MS and MS + Enr groups. In MDD patients, plasma miR-132 expression is elevated and is also reduced by escitalopram treatment [61]. Let-7 g-3p was another hub miRNA that was significantly downregulated by MS. The let-7 family of miRNAs has been strongly implicated in various aspects of MDD pathophysiology [62]. Between various hub miRNAs, several common gene targets were found including PTEN, MAPK6, CALM1, WNT2B, and GRIN2B.

From the list of most commonly target genes, we selected several genes for qCPR-based validation. GABRA1 (GABA receptor subunit alpha-1) showed increased expression in both MS and MS + Enr groups. MAPK6 (mitogen-activated protein kinase 6) and MMP19 (matrix metalloproteinase-19) showed increased expression in MS, but MS + Enr groups showed expression reversal. One study found that GABRA1 was downregulated in postmortem brain of MDD patients who died by suicide [63]. Our findings only show increased expression of WNT2B in MS + Enr animals compared to controls, though not in MS animals without Enr. Wnt family proteins act on the ß-catenin pathway and may play a role in mood disorder pathophysiology [64]. Our group recently found decreased WNT5B expression associated with increased miR-128 in rodents with learned helplessness [65]. Another study found that neonatal MS reduced myelination in the prefrontal cortex via Wnt signaling changes [66].. MAPK6 encodes ERK3 and is widely expressed in the brain. ERK3 plays an important role in neonatal growth [67]. MAPK pathways have also been found to regulate some MMPs [68]. Wojtowicz and Mozrzymas [69] found that FN-439, a synthetic compound which blocks active MMP sites via chelation of Zn^2+^, led to a loss of long-term potentiation in CA1 of the hippocampus. However, there is currently little research specific to MMP19.

All 4 of these genes are targeted by miR-301b-3p. Following the canonical pattern of expression between miRNAs and their targets, miR-301b-3p was significantly downregulated while all 4 genes were upregulated. Furthermore, MS + Enr animals showed miR-301b expression more similar controls—the opposite as seen in MAPK6 and MMP19 expression. miR-144 was also a targeting miRNA for both MMP19 and MAPK6. This is one of the first studies to support coregulation of these pathways by miRNAs. Furthermore, they may respond to Enr as a preventative for depressive behavior, particularly anhedonia. In our study, Enr was associated with significant improvement in several behavior. Sucrose preference levels in MS + Enr animals were closer to that of control animals, showing the potential for Enr to protect against the depressogenic effects of MS. Enr animals also showed significantly increased total movement in the EPM indicating a reduced anxiety phenotype. Reduced adrenal weight in Enr animals suggests that Enr reduced engagement of the HPA stress axis, although CORT levels were unchanged.

We identified CPG islands near 4 miRNA promoter regions. miR-219a showed a significant increase in relative methylation following MS. Following the canonical relationship between methylation and decreased gene transcription [70], miR-219 was significantly downregulated in both MS and MS + Enr cohorts. This is the first study to implicate methylation as a potential mechanism of miRNA regulation following MS. TGFBR2 and ESR1 (Estrogen receptor 1) are both gene targets of miR-219a; however, neither showed significant upregulation following MS. A study of environmental enrichment in rats found increased expression of miR-219 in serum exosomes [71]. This study also transfused the serum of young, enriched animals into aging animals and found evidence of increased oligodendrocyte development. Further research is needed to parse the interaction between miRNA expression, miRNA methylation, estrogens, and environmental enrichment.

## Conclusions

Both ELS and acute stress have widespread effects on miRNA expression which may mediate changes in stress-related behaviors. We found that sex significantly altered miRNA expression in response to stress, often with male animals showing more robust and extensive changes. This effect may be due to pre-pubertal timing of MS and highlights the need for more detailed characterization of the effect of ELS timing on behavior and neurobiology. Furthermore, this study identified several hypothalamic miRNAs of interest with regards to ELS. It is important for future studies to test whether direct manipulation of these candidate miRNAs lead to or even prevent later stress susceptibility and whether these behavioral changes are immediate and/or long-lasting. Enrichment had a particularly strong effect on animal behavior and miRNA expression and even reversed some of the effects of MS especially in the MAPK signaling pathway. These findings highlight enrichment as a noninvasive means of altering miRNAs which could prove useful in treating or preventing MDD onset. Finally, increased methylation may mediate some of the changes in miRNA expression resulting from ELS. Future studies utilizing next-generation sequencing will be crucial to detecting methylation of miRNAs with better resolution.

## Methods

### Animals

The study was approved by the Institutional Animal Care and Use Committee at the University of Alabama at Birmingham and was performed in accordance with relevant regulations, including ARRIVE guidelines. Animals were housed under standard care conditions (ad libitum food and water, 27 °C, 12-h light-dark cycle) for the duration of the experiment. Each group included 6 animals. The expanded methods are described in Supplementary File 1.

### Maternal separation

An overview of the experiment is shown in Fig. 1a**.** Each litter of Holtzman rats (Envigo, Indianapolis, IN, USA) was randomly assigned to either the control (non-MS) or MS group. Non-MS controls were handled for 5 min from PND 1–14. MS pups were separated from the dam and housed individually on a 33 °C heating pad for 180 min each morning until PND 14. Pups were weaned from the dam on PND 21.

### Restraint stress

Animals from both control and MS groups were randomly assigned to RS or control groups (non-RS). At PND 80, RS animals were placed in 20 cm restrainer tubes for 120 min each day for 7 days. Non-RS controls were handled but not restrained.

### Environmental enrichment

A subset of MS and MS + RS animals was randomly assigned to receive Enr from PND 21 to 90. Enrichment included colored toys, tubes, shreddable cotton and paper objects, and manzanita wood which were rotated weekly to maintain novelty. Non-enriched animals were housed conventionally.

### Animal behavior

#### Sucrose preference test

Sucrose preference was tested immediately following RS to assess anhedonia as previously described [72]. Briefly, on day 1, the animals were given sucrose1% (w/v). After acclimation on day 2, the animals were given access to both regular water and sucrose. Then the animals fasted for 24 h. On day 4, the animals were housed individually and given a premeasured 500 mL bottle of 1% sucrose and regular water. After 8 h, each of the bottles was measured for consumption and sucrose preference was calculated as previously described [72].

#### Elevated plus maze

Animals were placed individually in the center of a raised plus-shaped platform (50 × 50 cm) with two open and two walled arms (15 cm tall, open roof). For 5 min, the animals were recorded using Noldus Ethovision XT 11.5. Open and closed arm time and frequency were recorded and anxiety index was calculated as published earlier [73].

#### Forced swim test

Animals were acclimated for 15-min to an acrylic cylinder (28 cm diameter × 46 cm tall) filled to 25 cm with room temperature water. 24 h later, each animal was recorded in the same swim condition for 6 min and was returned to their home cage. A rater blinded to the conditions scored the videos using Kinoscope [74].

#### Shuttle escape test

As reported previously [72], escape latency was tested using a two-chamber shuttlebox with an electrified grid floor (70 cm × 20 cm × 20 cm, Med Associates, IN, USA). For the initial 5 trials, foot shocks were delivered on a variable interval schedule (0.6 mA, 60 s average interval) and terminated when the animal crossed into the opposite chamber. For the next 25 trials, the foot shocks terminated after the animal crossed into the opposite chamber and back. Escape latency was recorded by a PC connected to the shuttle box and shock generator.

### Vaginal cytology, tissue collection, and RNA isolation

The morning following escape testing, prior to tissue collection, female animals were tested for estrous phase by vaginal lavage. A blinded-rater assessed the samples according to cell morphology as previously described [75, 76]. The animals were anesthetized with isofluorane and blood was collected via cardiac puncture. The brain and adrenal glands were dissected, flash frozen, and stored at − 80 °C until further use. RNA was extracted from hypothalamus using TRIzol (Invitrogen, NY, USA) as described in Roy, Dunbar [34]. RNA concentration and quality were tested using Nanodrop 2000C Spectrophotometer; 260/280 nm > 1.7 was considered pure.

### Corticosterone, estradiol, and progesterone ELISAs

CORT, Estradiol, and Progesterone were quantified in platelet-free plasma using enzyme-linked immunosorbent assay (ELISA) (Enzo Life Sciences, NY, USA).

### miRNA sequencing

RNA sequencing libraries were prepared using the Qiaseq miRNA library kit (Qiagen, Hilden, Germany) and were sequenced on a NextSeq 500 (Illumina, CA, USA). Data were extracted using Qiagen Gene Globe and CPM was calculated using *edgeR.*

### Statistical approach

A 2 × 2 × 2 ANOVA was conducted in *R* to examine behavior and miRNA expression differences between MS and RS groups as well as sex. We also conducted a 2 × 2 × 2 ANOVA to examine the interaction of RS, sex, and Enr within MS animals on behavior and miRNA expression. Figure 1b is a visual representation of the animals/groups which were used in each analysis. We used a 1-way ANOVA to compare control, MS, and MS + Enr group behavior and miRNA expression. Subsequent bioinformatic analysis and follow-up gene expression and methylation studies were based on miRNA expression changes found when comparing control, MS, and MS + enrichment groups. To determine if sex played a role in MS and Enr-related gene target expression, we conducted a 2-way ANOVA. Only 1 gene showed a significant interaction, so the remaining gene expression comparisons were based on pairwise t-tests across group (control, MS, or MS + Enr). Group differences in qPCR-based methylation were tested using pairwise t-tests.

### Bioinformatic analysis

#### Chromosomal localization

miRbase.org was used to identify the chromosomal loci for significantly altered miRNAs. The Phenogram application (Ritchie Lab, University of Pennsylvania, USA) was used to plot the location of miRNAs across the rat karyotype.

#### Gene target prediction, miRNA-gene target networks, and gene ontology

A primary aim was to determine how MS leads to depression-like behavior via miRNA-based gene regulation. Thus, IPA was used to identify validated and highly predicted gene targets of miRNAs which were significantly altered by MS. This gene-target list was filtered based on each gene’s membership in canonical stress-related pathways. Futhermore, in order to identify a set of miRNAs with the greatest potential impact across these pathways only those pathways with > 16 gene targets were included. Because miRNAs can target many different genes and each gene may also be targeted by many miRNAs [26], pathways targeted by fewer miRNAs and with fewer compatible gene targets are less likely to be functionally affected by changes in an individual miRNA’s expression; it is possible that changes in a single miRNA may be negated by the activity of other miRNAs or even other regulatory mechanism. However, when several miRNAs targeting many genes within the same molecular pathway are altered, it is less likely that this cumulative effect will be countered by another miRNA or other mechanism. The identified miRNA targets were narrowed to 99 genes involved in Axonal Guidance Signaling (27 genes), Glucocorticoid Receptor Signaling (27 genes), Neuroinflammation Signaling (26 genes), Synaptogenesis Signaling (23 genes), Estrogen Receptor Signaling (23 genes), Protein Kinase A Signaling (19 genes), AMPK Signaling (18 genes), and ERK/MAPK Signaling (17 genes). miRNA-gene target networks were created using these 99 genes and significantly altered miRNAs based on one-way ANOVA. Among these miRNA-gene targeting relationships, miRNAs with more than 10 gene targets were identified as gene regulatory hubs and were also visualized in IPA as a miRNA-gene network. ShinyGO v0.61 [77] was used to explore GO associated with miRNA target genes. Metascape [78] was also used to confirm our GO analysis and to cluster ontologies with similar gene components.

Gene targets for qPCR follow-up were selected from the miRNA hub-gene target network based on their number of miRNA targets. Gene targets with the highest fold change as well as those which showed reversal of expression changes after Enr were also selected for qPCR-based expression testing.

### CDNA synthesis and gene target expression by qPCR

1 μg of RNA was reverse transcribed using an oligo dT priming method to synthesize first strand complimentary DNA (cDNA). Relative gene abundance was tested using qPCR and BrightGreen chemistry (Applied Biological Material, Canada). Primer sequences for the selected genes are listed in Supplementary Table 4. Livak’s ΔΔ CT [79] was method to quantify group differences. The data was normalized to the geometric mean of GAPDH, ß-actin, and 18srRNA. Because there was little variability in the housekeeping genes, each sample was tested individually for each gene of interest.

### Methylated DNA immunoprecipitation (MeDIP) and miRNA promoter region methylation by qPCR

Using the UCSC rodent genome browser tool, we searched for CPG islands near the promoter region of significantly altered miRNAs. We only found CPG islands upstream of 5 miRNAs and designed primers (Supplementary Table 4) to target these regions using Primer3 (v0.04.0; Untergasser, Cutcutache [80]). Genomic DNA (gDNA) was isolated from hypothalamus using phenol: chloroform: isoamylalcohol (25:24:1 V/V) chemistry. gDNA was sheared and immunoprecipitated with 5-methyl cytosine antibody (Zymo Research, CA). BrighGreen-based qPCR was used to quantify relative methylation enrichment in the immunoprecipitated DNA using the designed primers. Because of limited tissue availability, miRNA promoter methylation was tested in 48 of 72 original samples (*n* = 4 per group, including males and females).

## Supplementary Information

**Additional file 1.**

## Data Availability

The datasets generated and analyzed during the current study are available in the NCBI Sequence Read Archive (SRA) repository, at http://www.ncbi.nlm.nih.gov/bioproject/728805 and accession #: PRJNA728805.

## Abbreviations

ELS: early life stress
MDD: major depressive disorder
MS: maternal separation
RS: restraint stress
HPA: hypothalamic pituitary adrenal
CRH: corticotropin releasing hormone
ACTH: adrenocorticotropin releasing hormone
CORT: corticosterone
miRNA, miR: microRNA
RISC: RNA-induced silencing complex
Enr: environmental enrichment
EPM: elevated plus maze
FST: forced swim test
p/d: proestrus/diestrus
e/m: estrus/metestrus
CPM: counts per million
GO: gene ontology
cDNA: complimentary DNA
MeDIP: methylated DNA immunoprecipitation
