# Supplementary material for: Early life and adult stress promote sex dependent changes in hypothalamic miRNAs and environmental enrichment prevents stress-induced miRNA and gene expression changes in rats
Source: BMC Genomics. 2021 Sep 28;22:701. doi: 10.1186/s12864-021-08003-4 (PMC8480023; doi:10.1186/s12864-021-08003-4)
Supplement: Supplementary file 1 — Additional file 1. [file 12864_2021_8003_MOESM1_ESM.docx]

**Supplementary File 1**

**Early life and adult stress promote sex dependent changes in hypothalamic miRNAs and environmental enrichment prevents stress-induced miRNA and gene expression changes in rats**

^1^Lauren Allen McKibben, Ph.D. and ^1^Yogesh Dwivedi, Ph.D.*

^1^Department of Psychiatry and Behavioral Neurobiology

University of Alabama at Birmingham

Birmingham, Alabama, USA

**Running Title:** Sex-dependent early-life stress and hypothalamic miRNAs

**Keywords:** Early life stress, microRNA, hypothalamus, restraint stress, enrichment, microRNA sequencing, methylation, rat

***Corresponding Author**

Yogesh Dwivedi, Ph.D.

Elesabeth Ridgely Shook Professor

Director of Translational Research, UAB Mood Disorder Program

Co-Director, UAB Depression and Suicide Center

Department of Psychiatry and Behavioral Neurobiology

University of Alabama at Birmingham

SC711 Sparks Center

1720 7^th^ Avenue South

Birmingham, Alabama, USA

Email: [ydwivedi@uab.edu](mailto:ydwivedi@uab.edu)

**Methods**

***Maternal Separation****.* Pregnant Holtzman rats (Envigo, Indianapolis, IN, USA) were monitored for pups, twice daily beginning on gestational day 20. Once born, the whole litter was randomly assigned to either the control (non-MS) or maternal separation (MS) group. Non-MS controls were handled for 5 minutes each morning from postnatal day 1-14 and were then immediately returned to their home cage. MS pups were separated from the dam and other pups in the litter and housed individually on a 33°C heating pad for 180 minutes each morning until PND 14. MS-180 and control animals were each comprised from several litters to reduce potential litter effects. After PND 14, pups were housed with the dam until weaning at PND 21.

***Restraint Stress.*** At PND 21, animals from both control and MS groups were randomly assigned to restraint stress (RS) or control groups (non-RS) and housed in same-sex groups of 2-4 based on their assigned experimental group. At PND 80, RS animals were placed in 20 cm restrainer tubes for 2 hours each day for 7 days. Non-RS controls were exposed to the tubes and handled but were not restrained. Following RS, we began testing for depression-like behavior.

***Environmental Enrichment.*** A subset of MS animals was randomly assigned to receive environmental enrichment (Enr) from PND 21 to 90. Enrichment consisted of added colored toys, tubes, shreddable objects, and manzanita wood within standard housing. Enrichment objects were rotated weekly in order to maintain novelty. This enrichment montage was chosen based on findings of previous studies implementing similar interventions (1-4). Those animals assigned to no enrichment were housed in conventional housing. MS animals with enrichment were also randomized to either control (non-RS) or RS (Enr+RS).

***Behavior Data Collection***

All animals were tested using the Sucrose Preference Test (SPT), Elevated Plus Maze (EPM), Forced Swim Test (FST), and Shuttle Escape Test (SET).

***Sucrose Preference Test.*** Animals were administered the SPT in order to test for anhedonia as previously described (5). Briefly, on day 1, the animals were provided with 1% sucrose water. After acclimation on day 2, the animals were given access to both regular water and sucrose water. Then the animals fasted for 24 hours. On day 4, the animals were housed individually and given a premeasured 500 mL bottle of 1% sucrose and regular water. After 8 hours, each of the bottles was measured for consumption and sucrose preference was calculated as previously published (5).

***Elevated Plus Maze.*** Animals were placed individually in the center of a raised plus-shaped platform (50x50 cm) with two open and two walled arms (15 cm tall, open roof). For 5 minutes, the animals were recorded using Noldus Ethovision XT 11.5. Open and closed arm time and frequency were extracted using the software. We also calculated an anxiety index score as published by Cohen, Matar (6).

***Forced Swim Test.*** Animals were acclimated for 15-minutes to an acrylic cylinder (28 cm diameter x 46 cm tall) filled to 25cm of room temperature water. 24 hours later, each animal was placed in the same cylinder filled with clean room temperature water for 6 minutes. We recorded the animals using a camcorder and a blind rater scored the videos using Kinoscope (7). Following the test, each animal was towel dried and returned to their home cage.

***Corticosterone, Estradiol, and Progesterone ELISAs.*** Blood was processed into fractions immediately following collection. Platelet-free plasma was collected by centrifugation. Blood components were stored at -80°C.

Corticosterone (CORT), Estradiol, and Progesterone were quantified in plasma using enzyme-linked immunosorbent assay (ELISA) kits from Enzo Life Sciences (Farmingdale, NY, USA). Plasma concentrations were optimized prior to testing; for CORT we used a 1:40 dilution, for Estradiol we used 1:15 dilution, and for Progesterone we used a 1:200 dilution. Corticosterone was tested in order to validate the stress-inducing effect of MS and RS. Estradiol and Progesterone levels were tested to validate the estrous phase ratings based on vaginal cytology.

***Sequencing Analysis***

We conducted group comparisons of CPM expression values using *R.* A 2 x 2 x 2 ANOVA was conducted to examine differences between MS and RS groups as well as sex. We also conducted a 2 x 2 x 2 ANOVA to examine the interaction of RS, sex, and Enr within MS animals. Finally, we used a 1-way ANOVA to compare control, MS, and MS+Enr groups. Subsequent bioinformatic analysis and follow-up gene expression and methylation studies were based on miRNA expression changes found when comparing control, MS, and MS+enrichment groups.

***Bioinformatic Analysis***

***Gene target prediction.*** Ingenuity Path Analysis (IPA; Qiagen, Hilden Germany) was used to determine experimentally validated and highly predicted gene targets of those miRNAs altered by maternal separation. This gene target list was filtered to include genes involved in top canonical pathways (>16 genes from full list) related to depression. Expression of these genes was quantified using qPCR.

***Gene ontologies.*** ShinyGO v0.61 (8) was used to explore gene ontologies (GO) associated with our filtered gene list. We extracted the top 20 ontologies for KEGG Pathways, Molecular Function, Cellular Component, and Biological Processes. Metascape (9) was also used to confirm our GO analysis. Metascape selects the top ontologies from all 4 GO categories rather than separately. Metascape also uses hierarchical clustering to determine how closely related each ontology is to another and maps this as an ontology network.

***CDNA Synthesis and Gene Target Expression by qPCR***

One μg of hypothalamus RNA was reverse transcribed using an oligo dT priming method to synthesize first strand complimentary DNA (cDNA). We tested for relative gene abundance using quantitative PCR and BrightGreen chemistry (Applied Biological Material, Richmond, Canada). We selected depression and stress related genes based on literature from the gene targets of miRNAs significantly altered by MS and designed primers using PrimerBlast (NCBI, Bethesda, MD, USA). Primer sequences are listed in **Supplementary Table 6**. We used the ΔΔ CT method to quantify group differences in relative expression. Genes of interest were normalized to the geometric mean of CT values for GAPDH, ß-actin, and 18srRNA.

*Methylated DNA Immunoprecipitation (MeDIP) and miRNA Promoter Region Methylation by qPCR*

Using the UCSC rodent genome browser tool, we searched for CPG islands—potential methylation sites—near the promoter region of significantly altered miRNAs. We identified CPG islands upstream of 5 miRNAs and designed primers (**Supplementary Table 4)** to target these regions using Primer3 (v0.04.0; Untergasser, Cutcutache (10)). Genomic DNA (gDNA) was isolated from 5 mg of hypothalamus tissue using phenol: chloroform: isoamylalcohol (25 : 24 : 1 V/V) chemistry. Briefly, 5 μg of total gDNA was treated with RNaseA and sonicated to generate a random fragment length of ~400 bp. We denatured the gDNA and then conjugated it using PureProteome Protein A/G Mix Magnetic Beads (EMD Millipore) and 5 μg monoclonal 5-methyl cytosine antibody (Zymo Research, Irvine, CA). 10% of the untreated sonicated denatured gDNA was saved as input control. Following immunoprecipitation (IP), DNA was extracted from both IP and input samples, again using phenol: chloroform: isoamylalcohol (25 : 24 : 1 V/V) method. We used BrightGreen-based qPCR on the MeDIP DNA to amplify CPG islands using the previously mentioned primers. We normalized the methylation enrichment CT values with the input control for analysis. Because of limited tissue available for DNA synthesis, we tested for miRNA promoter methylation in 61 of 72 original samples, 19 controls, 20 MS, and 22 MS+Enr.

**Results**

***The effect of estrus on behavior and miRNAs***

We dichotomized the estrus phase into proestrus/diestrus (p/d) and estrus/metestrus (e/m) because of their expected Estradiol and Progesterone levels. **Supplementary Figure 1a-d** shows representative microscope images of vaginal cell cytology which was used to identify estrus phase at the time of sacrifice. We found that 21 animals were in p/d and 15 were in e/m. The e/m group consisted of 80% MS animals whereas the p/d group only consisted of 57% MS animals. The p/d group also consisted largely of non-enriched animals (81%). The results of our progesterone ELISA confirmed the blind ratings of estrus phase; p/d animals had higher (though not significant; t(34)=1.92, *p*=0.064) progesterone levels than those animals sacrificed during the e/m phase (**Supplementary Figure 1e)**. Estradiol levels were similar across groups with the e/m animals having slightly higher plasma levels (**Supplementary Figure 1f**). There were no significant estrus-based differences in plasma CORT, adrenal weight, or body weight at the time of sacrifice (**Supplementary Figure 1g-i**). Of the tested behaviors, only FST showed differences in behavior based on estrus phase; animals in p/d exhibited significantly lower swim scores (p/d M=38.10± SEM=3.11, e/m M=48.07±3.32, t(34)=-2.16, *p*<0.05) and higher climb scores (p/d M=14.62±1.18, e/m M=7.80±1.72, t(34)=3.38, *p*<0.005) than e/m animals. These differences were expected based on the rate of MS animals in each of the estrus phase groups. We then tested for a significant difference in miRNA expression due to estrus phase. Only 6 miRNAs were significantly associated with estrus phase (**Supplementary Table 5**), including miRs-28-5p, -155-5p, -362-3p, -374-5p, -384-5p, and -872-5p.

***Gene ontologies associated with maternal separation***

The top KEGG pathways related to brain function (**Supplementary** **Figure 2a**) were ras signaling pathway (19 genes), EGFR tyrosine kinase inhibitor resistance (13 genes), and neurotrophin signaling pathway (14 genes). MAPK signaling (16 genes) and estrogen signaling (12 genes) were also among the top 20. The top molecular functions (**Supplementary** **Figure 2b**) were ion binding (43 genes), enzyme binding (32 genes), and catalytic activity acting on a protein (29 genes). The cellular component ontologies (**Supplementary** **Figure 2c**) non-membrane bound organelle (39 genes), intracellular non-membrane bound organelle (38 genes), and neuron part (33 genes) included the greatest number of target genes. The top biological process ontologies (**Supplementary** **Figure 2d**) were anatomical structure development (57 genes), multicellular organism development (56 genes), and system development (54 genes). Metascape’s GO analysis revealed pathways in ras signaling, positive regulation of cell development, response to extracellular stimulus, and negative regulation of intracellular signal transduction as the top overall ontologies (**Figure 2c**). **Figure 2d** shows the clustering of ontologies based on similar member genes. Colored nodes in the top map of **Figure 2d** represent the top ontologies as listed in the legend. Node colors in bottom map of **Figure 2d** represent the significance of hierarchical clustering.

**References**

1. Durairaj RV, Koilmani ER. Environmental enrichment modulates glucocorticoid receptor expression and reduces anxiety in Indian field male mouse Mus booduga through up-regulation of microRNA-124a. Gen Comp Endocrinol. 2014;199:26-32.

2. Sztainberg Y, Kuperman Y, Tsoory M, Lebow M, Chen A. The anxiolytic effect of environmental enrichment is mediated via amygdalar CRF receptor type 1. Mol Psychiatry. 2010;15(9):905-17.

3. Morley-Fletcher S, Rea M, Maccari S, Laviola G. Environmental enrichment during adolescence reverses the effects of prenatal stress on play behaviour and HPA axis reactivity in rats. Eur J Neurosci. 2003;18(12):3367-74.

4. Francis DD, Diorio J, Plotsky PM, Meaney MJ. Environmental enrichment reverses the effects of maternal separation on stress reactivity. The Journal of neuroscience : the official journal of the Society for Neuroscience. 2002;22(18):7840-3.

5. Timberlake Ii M, Roy B, Dwivedi Y. A Novel Animal Model for Studying Depression Featuring the Induction of the Unfolded Protein Response in Hippocampus. Mol Neurobiol. 2019;56(12):8524-36.

6. Cohen H, Matar MA, Joseph Z. Animal models of post-traumatic stress disorder. Curr Protoc Neurosci. 2013;Chapter 9:Unit 9 45.

7. Kokras N, Baltas D, Theocharis F, Dalla C. Kinoscope: An Open-Source Computer Program for Behavioral Pharmacologists. Front Behav Neurosci. 2017;11:88.

8. Ge SX, Jung D, Yao R. ShinyGO: a graphical gene-set enrichment tool for animals and plants. Bioinformatics. 2020;36(8):2628-9.

9. Zhou Y, Zhou B, Pache L, Chang M, Khodabakhshi AH, Tanaseichuk O, et al. Metascape provides a biologist-oriented resource for the analysis of systems-level datasets. Nat Commun. 2019;10(1):1523.

10. Untergasser A, Cutcutache I, Koressaar T, Ye J, Faircloth BC, Remm M, et al. Primer3--new capabilities and interfaces. Nucleic Acids Res. 2012;40(15):e115.

**Supplementary Tables and Figures**

**Supplementary Figure 1.** Representative vaginal cytology and sex hormone levels


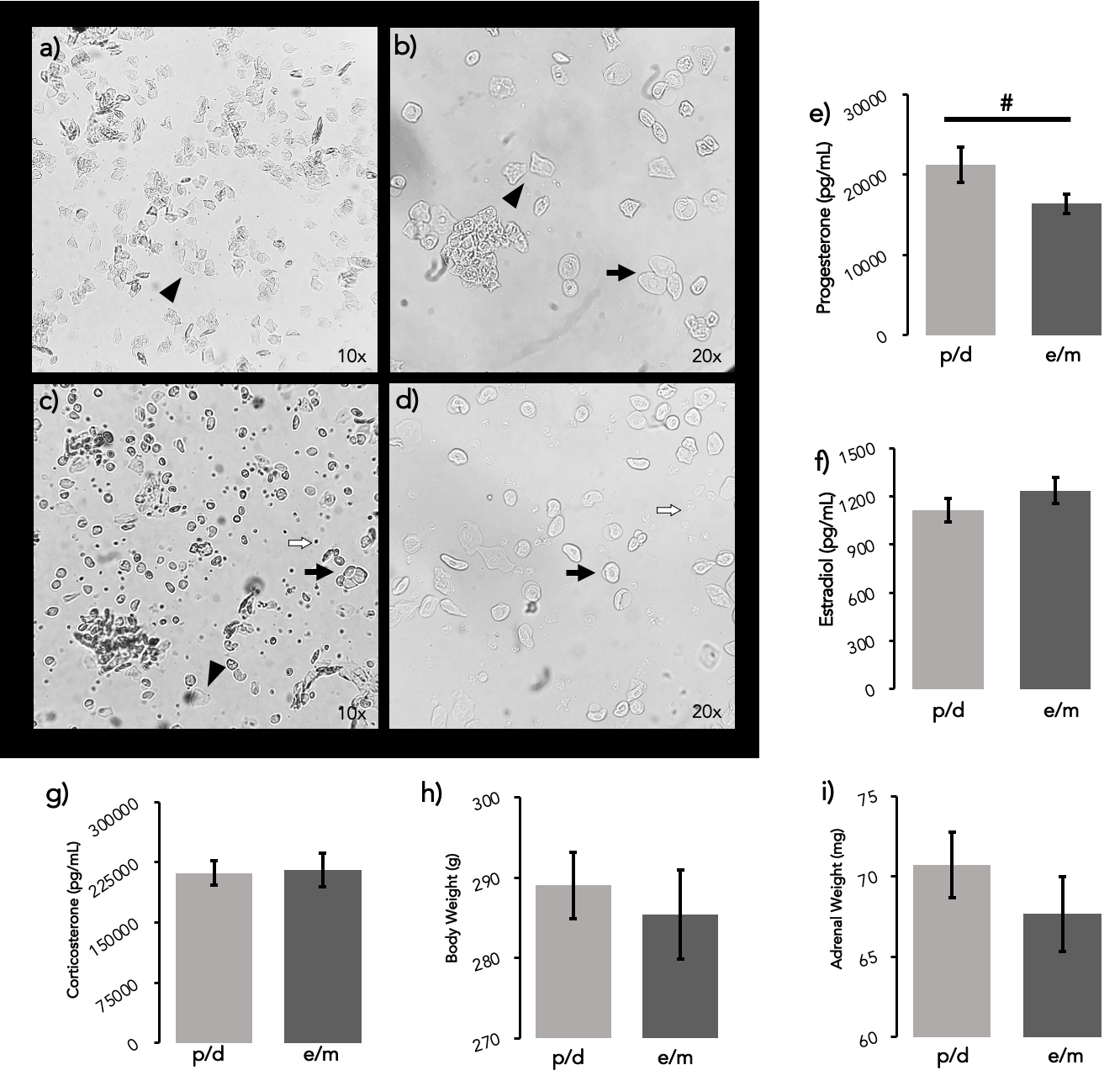


**Supplementary Figure 1.** Representative vaginal Cytology and Sex Hormone Levels

The type and ratio of cell types from vaginal lavage were used to determine estrous phase upon sacrifice. Representative images of estrus and/or proestrus at **a)** 10x and **b)** 20x magnification have very few leukocytes present. Representative images of Metestrus and/or Diestrus at **c)**10x and **d)** 20x magnification have a larger number of leukocytes present. Black arrows in **b, c**, and **d** show nucleated epithelial cells. Black arrowheads in **a, b**, and **c** show cornified epithelial cells and the white arrows in **c** and **d** show leukocytes. **e)** Animals in proestrus/diestrus phase exhibited a trending increase in progesterone level but **f)** estradiol levels were not different between estrus phases. Physiological measures of stress such as **g)** corticosterone level, **h)** body weight, and **i)** adrenal weight were not affected by estrus phase. *Abbreviations: p/d-proestrus/diestrus, e/m-estrus/metestrus.*

|  | **Supplementary Table 1.** miRNAs significantly altered by maternal separation and enrichment in males | | | | | | | |  |
| --- | --- | --- | --- | --- | --- | --- | --- | --- | --- |
|  |  |  |  | **CPM-Fold change** | | | *p (one-way ANOVA)* |  |  |
|  |  | Accession ID | miRNA | MS/C | MS+Enr/C | MS+Enr/MS |  | *sig.* |  |
|  |  |  |  |  |  |  |  |  |  |
|  | *upregulated by MS* | |  |  |  |  |  |  |  |
|  |  | *MIMAT0000838* | *miR-132-3p* | 1.449 | 1.567 | 1.081 | *0.000* | *a,b,d,f* |  |
|  |  | *MIMAT0000859* | *miR-181b-5p* | 1.351 | 1.051 | -1.286 | *0.024* | *b,d,* |  |
|  |  | *MIMAT0017304* | *miR-411-3p* | 1.351 | 1.051 | -1.286 | *0.022* | *a,d,e,* |  |
|  |  | *MIMAT0000550* | *miR-323-3p* | 1.337 | 1.322 | -1.012 | *0.001* | *a,b,d,f* |  |
|  |  | *MIMAT0035730* | *miR-1843b-5p* | 1.337 | 1.322 | -1.012 | *0.014* | *a,b,d,* |  |
|  |  | *MIMAT0017158* | *miR-212-5p* | 1.306 | 1.611 | 1.234 | *0.004* | *b,d,f* |  |
|  |  | *MIMAT0017334* | *miR-146b-3p* | 1.306 | 1.611 | 1.234 | *0.017* | *a,b,d,* |  |
|  |  | *MIMAT0000822* | *miR-100-5p* | 1.295 | 1.429 | 1.104 | *0.001* | *b,c,d,f* |  |
|  |  | *MIMAT0003205* | *miR-409a-3p* | 1.282 | 1.420 | 1.108 | *0.000* | *a,b,c,d,f* |  |
|  |  | *MIMAT0003379* | *miR-378a-3p* | 1.227 | 1.307 | 1.065 | *0.012* | *b,d,f* |  |
|  |  | *MIMAT0005299* | *miR-181d-5p* | 1.224 | 1.650 | 1.348 | *0.041* | *a,b,d,* |  |
|  |  | *MIMAT0001626* | *miR-431* | 1.223 | 1.495 | 1.223 | *0.003* | *a,b,d,f* |  |
|  |  | *MIMAT0017163* | *miR-221-5p* | 1.217 | 1.558 | 1.280 | *0.004* | *b,d,f* |  |
|  |  | *MIMAT0000820* | *miR-99a-5p* | 1.184 | 1.288 | 1.087 | *0.000* | *b,c,d,f* |  |
|  |  | *MIMAT0003177* | *miR-541-5p* | 1.174 | 1.309 | 1.115 | *0.045* | *b,d,f* |  |
|  |  | *MIMAT0005321* | *miR-500-3p* | 1.169 | 1.400 | 1.197 | *0.046* | *b,d,f* |  |
|  |  | *MIMAT0017819* | *miR-3557-5p* | 1.167 | 1.359 | 1.165 | *0.030* | *b,d,f* |  |
|  |  | *MIMAT0004706* | *let-7e-3p* | 1.161 | 1.298 | 1.118 | *0.006* | *a,b,d,f* |  |
|  |  | *MIMAT0003202* | *miR-382-3p* | 1.154 | 1.530 | 1.326 | *0.014* | *b,d,f* |  |
|  |  | *MIMAT0004716* | *miR-28-3p* | 1.143 | 1.177 | 1.030 | *0.032* | *b,d,f* |  |
|  |  | *MIMAT0005282* | *miR-872-5p* | 1.143 | 1.399 | 1.224 | *0.036* | *b,d,* |  |
|  |  | *MIMAT0017870* | *miR-409b* | 1.143 | 1.467 | 1.284 | *0.006* | *b,d,f* |  |
|  |  | *MIMAT0003204* | *miR-409a-5p* | 1.139 | 1.182 | 1.037 | *0.016* | *b,d,f* |  |
|  |  | *MIMAT0000574* | *miR-140-3p* | 1.135 | 1.298 | 1.144 | *0.004* | *b,c,d,f* |  |
|  |  | *MIMAT0024843* | *miR-1839-5p* | 1.113 | 1.288 | 1.157 | *0.017* | *b,d,f* |  |
|  |  | *MIMAT0000583* | *miR-339-5p* | 1.108 | 1.353 | 1.221 | *0.015* | *b,d,f* |  |
|  |  | *MIMAT0004710* | *miR-17-1-3p* | 1.106 | 1.317 | 1.190 | *0.004* | *b,c,d,f* |  |
|  |  | *MIMAT0035719* | *let-7g-5p* | 1.036 | 1.240 | 1.196 | *0.017* | *b,d,f* |  |
|  |  |  |  |  |  |  |  |  |  |
|  | *downregulated by MS* | |  |  |  |  |  |  |  |
|  |  | *MIMAT0000850* | *miR-144-3p* | -2.301 | -3.375 | -1.467 | *0.001* | *a,b,d,f* |  |
|  |  | *MIMAT0003115* | *miR-207* | -1.680 | -1.377 | 1.220 | *0.039* | *a,d* |  |
|  |  | *MIMAT0017886* | *miR-3596a* | -1.446 | -1.131 | 1.279 | *0.043* | *a,c,e* |  |
|  |  | *MIMAT0017839* | *miR-3065-5p* | -1.402 | -1.261 | 1.112 | *0.015* | *a,d,e* |  |
|  |  | *MIMAT0017230* | *miR-497-3p* | -1.377 | -1.860 | -1.351 | *0.007* | *b,d,f* |  |
|  |  | *MIMAT0017838* | *miR-218b* | -1.329 | -1.178 | 1.129 | *0.020* | *a,e* |  |
|  |  | *MIMAT0000812* | *miR-33-5p* | -1.313 | -2.323 | -1.769 | *0.001* | *b,c,d,f* |  |
|  |  | *MIMAT0017818* | *miR-3556b* | -1.310 | 1.036 | 1.357 | *0.017* | *a,c,e* |  |
|  |  | *MIMAT0017903* | *miR-3595* | -1.276 | -1.365 | -1.069 | *0.006* | *a,b,d,f* |  |
|  |  | *MIMAT0017219* | *miR-376c-5p* | -1.264 | -1.620 | -1.282 | *0.003* | *b,d,f* |  |
|  |  | *MIMAT0000801* | *miR-29b-3p* | -1.243 | -1.812 | -1.457 | *0.014* | *b,d,f* |  |
|  |  | *MIMAT0017143* | *miR-186-3p* | -1.238 | -1.456 | -1.176 | *0.016* | *b,d,f* |  |
|  |  | *MIMAT0017883* | *miR-3587* | -1.226 | -1.244 | -1.014 | *0.017* | *a,b,d* |  |
|  |  | *MIMAT0003197* | *miR-376a-5p* | -1.218 | -1.653 | -1.358 | *0.006* | *b,d,f* |  |
|  |  | *MIMAT0000787* | *miR-18a-5p* | -1.210 | -1.635 | -1.351 | *0.018* | *b,d,f* |  |
|  |  | *MIMAT0000842* | *miR-136-5p* | -1.204 | -1.824 | -1.515 | *0.009* | *b,c,d,f* |  |
|  |  | *MIMAT0035720* | *let-7g-3p* | -1.200 | -1.517 | -1.264 | *0.019* | *b,d,f* |  |
|  |  | *MIMAT0000805* | *miR-30e-5p* | -1.194 | -1.428 | -1.196 | *0.002* | *b,d,f* |  |
|  |  | *MIMAT0017888* | *miR-3589* | -1.188 | -1.208 | -1.017 | *0.036* | *a,b,d* |  |
|  |  | *MIMAT0017135* | *miR-153-5p* | -1.174 | -1.577 | -1.344 | *0.010* | *b,c,d,f* |  |
|  |  | *MIMAT0004707* | *let-7i-3p* | -1.172 | -1.315 | -1.122 | *0.003* | *b,c,d,f* |  |
|  |  | *MIMAT0004732* | *miR-135a-3p* | -1.171 | -1.668 | -1.425 | *0.005* | *b,d,f* |  |
|  |  | *MIMAT0003198* | *miR-376a-3p* | -1.167 | -1.323 | -1.134 | *0.005* | *b,d,f* |  |
|  |  | *MIMAT0000804* | *miR-30c-5p* | -1.164 | -1.365 | -1.172 | *0.016* | *b,d,f* |  |
|  |  | *MIMAT0005328* | *miR-673-5p* | -1.162 | -1.569 | -1.350 | *0.005* | *b,d,f* |  |
|  |  | *MIMAT0017026* | *miR-301a-5p* | -1.150 | -1.624 | -1.413 | *0.010* | *b,c,d,f* |  |
|  |  | *MIMAT0005596* | *miR-551b-3p* | -1.115 | -1.402 | -1.257 | *0.015* | *b,d,f* |  |
|  |  | *MIMAT0000815* | *miR-34a-5p* | -1.082 | -1.234 | -1.141 | *0.008* | *b,d,f* |  |
|  |  | ^a^ controls vs. MS |  |  |  |  |  |  |  |
|  |  | ^b^ controls vs. MS+Enr | |  |  |  |  |  |  |
|  |  | ^c^ MS vs. MS+Enr |  |  |  |  |  |  |  |
|  |  | ^d^ controls vs. MS and MS+Enr | |  |  |  |  |  |  |
|  |  | ^e^ controls and MS+Enr vs. MS (recovered by Enr) | | |  |  |  |  |  |
|  |  | ^f^ controls and MS vs. MS+Enr | |  |  |  |  |  |  |
|  |  |  |  |  |  |  |  |  |  |

Significant changes in miRNA expression after MS and Enr based on one-way ANOVA specifically in male animals are shown. Listed miRNAs are split by those upregulated vs. downregulated by MS. CPM-foldchange value for MS and Enr was calculated as a ratio between two group’s CPM expression. Positive values indicate increased expression and negative values show reduced expression in the numerator group. The full one-way ANOVA significance is listed as *p.* Specific group differences are listed as *sig; abbreviations: CPM-counts per million, MS-maternal separation, C-control, Enr-environmental enrichment.*

|  | **Supplementary Table 2.** miRNAs significantly altered by maternal separation and enrichment in females | | | | | | |  |  |
| --- | --- | --- | --- | --- | --- | --- | --- | --- | --- |
|  |  |  |  | **CPM-Fold change** | | | *p (one-way ANOVA)* |  |  |
|  |  | Accesion ID | miRNA | MS/C | MS+Enr/C | MS+Enr/MS |  | *sig.* |  |
|  |  |  |  |  |  |  |  |  |  |
|  | *upregulated by MS* | |  |  |  |  |  |  |  |
|  |  | *MIMAT0003378* | *miR-378a-5p* | 1.339 | -1.012 | -1.355 | *0.020* | *a,c,e* |  |
|  |  | *MIMAT0017158* | *miR-212-5p* | 1.201 | 1.585 | 1.320 | *0.005* | *a,b,d,f* |  |
|  |  | *MIMAT0017123* | *miR-132-5p* | 1.078 | 1.246 | 1.155 | *0.026* | *b,d,f* |  |
|  |  | *MIMAT0003194* | *miR-376c-3p* | 1.076 | 1.241 | 1.153 | *0.003* | *b,c,d,f* |  |
|  |  | *MIMAT0005299* | *miR-181d-5p* | 1.039 | 1.515 | 1.459 | *0.000* | *b,c,d,e,f* |  |
|  |  |  |  |  |  |  |  |  |  |
|  | *downregulated by MS* | |  |  |  |  |  |  |  |
|  |  | *MIMAT0017154* | *miR-206-5p* | -2.943 | -1.824 | 1.613 | *0.001* | *a,b,d,e* |  |
|  |  | *MIMAT0005445* | *miR-29b-1-5p* | -2.361 | -1.635 | 1.444 | *0.008* | *a,d,e* |  |
|  |  | *MIMAT0005304* | *miR-301b-3p* | -2.135 | -1.823 | 1.171 | *0.007* | *a,b,d,e* |  |
|  |  | *MIMAT0001543* | *miR-449a-5p* | -1.544 | -1.738 | -1.125 | *0.006* | *a,b,d,f* |  |
|  |  | *MIMAT0005328* | *miR-673-5p* | -1.256 | -1.256 | 1.000 | *0.034* | *a,b,d* |  |
|  |  | *MIMAT0000570* | *miR-331-3p* | -1.143 | -1.299 | -1.136 | *0.043* | *b,d* |  |
|  |  | *MIMAT0003207* | *miR-369-3p* | -1.001 | 1.298 | 1.299 | *0.001* | *b,c,d,f* |  |
|  |  | ^a^ controls vs. MS |  |  |  |  |  |  |  |
|  |  | ^b^ controls vs. MS+Enr |  |  |  |  |  |  |  |
|  |  | ^c^ MS vs. MS+Enr |  |  |  |  |  |  |  |
|  |  | ^d^ controls vs. MS and MS+Enr | |  |  |  |  |  |  |
|  |  | ^e^ controls and MS+Enr vs. MS (recovered by Enr) | | |  |  |  |  |  |
|  |  | ^f^ controls and MS vs. MS+Enr | |  |  |  |  |  |  |
|  |  |  |  |  |  |  |  |  |  |

miRNA expression after MS and Enr in female animals was tested using one-way ANOVA. Listed miRNAs are split by those upregulated vs. downregulated by MS. CPM-foldchange value for MS and Enr was calculated as a ratio between two group’s CPM expression. Positive values indicate increased expression and negative values show reduced expression in the numerator group. The full one-way ANOVA significance is listed as *p.* Specific group differences are listed as *sig; abbreviations: CPM-counts per million, MS-maternal separation, C-control, Enr-environmental enrichment.*

|  | **Supplementary Table 3.** miRNA and gene targets for expression testing | | | |  | |
| --- | --- | --- | --- | --- | --- | --- |
|  |  | gene | targeting miRNAs |  | |  |
|  |  |  |  |  | |  |
|  |  | GRIN2B | *miR-3557-5p, -132-3p, -181a-5p, -181b-5p, -181d-5p, -186-3p, -3065-5p, -331-3p, -34a-5p, -382-3p* |  | |  |
|  |  | SLC6A1 | *let-7g-3p, miR-132-3p, -212-5p, -221-5p, -34a-5p, -378a-3p, -425-5p* |  | |  |
|  |  | PTEN | let-7i-3p, miR-301a-5p, -301b-3p, -144-3p, -338-3p, -34a-5p, -425-5p |  | |  |
|  |  | TGFBR2 | miR-301a-5p, -301b-3p, -181a-5p, -181b-5p, -181d-5p, -219a-5p, -30e-5p, -338-3p, -34a-5p |  | |  |
|  |  | WNT2B | *miR-3557-5p, -301a-5p, -301b-3p, -181a-5p, -181b-5p, -181d-5p, -1839-5p, -331-3p, -34a-5p* |  | |  |
|  |  | MAPK6 | *let-7g-3p, miR-301a-5p, -301b-3p, -144-3p, -1839-5p, -425-5p* |  | |  |
|  |  | SMAD4 | *miR-301a-5p, -301b-3p, -17-1-3p, -1839-5p, -212-5p, -376c-3p* |  | |  |
|  |  | TSC22D3 | *miR-3557-5p, -132-3p, -132-5p, -186-3p, -425-5p* |  | |  |
|  |  | BCL2 | *let-7g-3p, miR-181a-5p, -181b-5p, -181d-5p, -34a-5p, -493-5p* |  | |  |
|  |  | CALM1 | *let-7g-3p, miR-301a-5p, -301b-3p, -181a-5p, -181b-5p, -181d-5p, -493-5p* |  | |  |
|  |  | FOXG1 | *let-7i-3p, miR-30e-5p, -34a-5p, -378a-3p* |  | |  |
|  |  | FOXO1 | *let-7g-3p, miR-378a-3p, -493-5p, -497-3p* |  | |  |
|  |  | PIK3CB | *miR-301a-5p, -301b-3p, -212-5p, -3065-5p, -34a-5p* |  | |  |
|  |  | ESR1 | *miR-301a-5p, -301b-3p, -181a-5p, -181b-5p, -181d-5p, -219a-5p* |  | |  |
|  |  | GABRA1 | *miR-301a-5p, -301b-3p, -144-3p, -181a-5p, 181b-5p, 181d-5p* |  | |  |
|  |  | GRM3 | *miR-132-3p, -376c-3p, -487b-3p* |  | |  |
|  |  | KALRN | *miR-301a-5p, -301b-3p, -323-3p, -431* |  | |  |
|  |  | GLI2 | *let-7i-3p, miR-378a-3p* |  | |  |
|  |  | MMP19 | *miR-301a-5p, -301b-3p, -144-3p* |  | |  |
|  |  | MMP9 | *miR-125b-2-3p, -132-3p* |  | |  |
|  |  | TBP | *let-7g-3p, miR-497-3p* |  | |  |
|  |  | TRAF6 | *miR-132-5p, -378a-3p* |  | |  |
|  |  | IL10RB | *miR-301a-5p, -301b-3p* |  | |  |
|  |  |  |  |  | |  |

| **Supplementary Table 4.** Forward and reverse qPCR primers | | | |
| --- | --- | --- | --- |
| Gene | | | Primer Sequence |
| BCL2 |  | F | GGACGCGAAGTGCTATTGGT |
| BCL2 |  | R | AGTATCCCACTCGTAGCCCC |
| CALM1 |  | F | CAGGAAACGGGTGAAGGCTA |
| CALM1 |  | R | CATGGGATTGGGGCACTTGA |
| FOXG1 |  | F | GAGGTGCAATGTGGGAGAAT |
| FOXG1 |  | R | CTGCACACATGGAAATCTGGC |
| GRIN2B |  | F | GGGTCACGCAAAACCCTTTC |
| GRIN2B |  | R | CCTTGTTTTTGACGCCCCTG |
| MAPK6 |  | F | GCGAAACCTAACCCACCCAT |
| MAPK6 |  | R | CATTGGTTACTGCGGCCAAC |
| SLC6A1 |  | F | CCTTCTTGCTGCTCGATGTTC |
| SLC6A1 |  | R | AGTTTGGGTGGAAATCAGGG |
| TGFBR2 |  | F | CCCCGTTTGGTTCCAGAGTG |
| TRFBR2 |  | R | GCACTCGGTCAGTGTCTCAC |
| TSC22D3 |  | F | CTGGAGCCACTTCCTTTCAAC |
| TSC22D3 |  | R | AGCTGTGGTTGGTTTGAGTG |
| WNT2B |  | F | GGGAGGCAGCGTTTGTCTAT |
| WNT2B |  | R | CCAGTCAAAGTCCCCTCGTT |
| FOXO1 |  | F | GGTAGGATGGGTGTCCTCCA |
| FOXO1 |  | R | TCTCCGTCCATGAGGTCGTT |
| GABRA1 |  | F | CTCTTATTCCCACACAGCCCA |
| GABRA1 |  | R | TTGCCCCTCTGAAAGCATCT |
| GLI2 |  | F | CTCCGCTCTTCGGCCTATTT |
| GLI2 |  | R | AGGCGAAAAAGCCAGAACCT |
| GRM3 |  | F | ATGCTGCTCCAGAACATGGAA |
| GRM3 |  | R | AGGGCACTAAAATCAGCCTAGAA |
| TBP |  | F | ACCTTTCCCACACAAGCAAGT |
| TBP |  | R | AGGCGGAATGTATCTGGCAC |
| TRAF6 |  | F | ACCATGATCCCTGGAAAGCTG |
| TRAF6 |  | R | TCAACAATGAAGGCAGGGGA |
| IL10RB |  | F | TGCTCACTAAAACAAGGACCAC |
| IL10RB |  | R | ATGGGGCCAGTTGCTTTTGA |
| KALRN |  | F | CCAATCGCACAGGAGAGGAG |
| KALRN |  | R | GGTTGAAGCTACCGACTGCT |
| SMAD4 |  | F | AGCCCTTCGTTTGCCATCA |
| SMAD4 |  | R | GTGGTACAGTCAATGCGTCC |
| PTEN |  | F | GCGTGCGGATAATGACAAGG |
| PTEN |  | R | TGGAGAGAAGTATCGGTTGGC |
| ESR1 |  | F | TCGAGCACATTCCTTCCTTCC |
| ESR1 |  | R | AGGTACAGATTGGCTTCCCG |
| MMP19 |  | F | GTCATTCCCCAGTCCCCATTT |
| MMP19 |  | R | CAACAATTTCCCTCCACCAGAA |
| MMP9 |  | F | GAGGAGCTAGTTTGCCGGAT |
| MMP9 |  | R | GCTGGTAAAGGTTGGGGGAT |
| PIK3CB |  | F | CTATGGCAGACACCCTTGACA |
| PIK3CB |  | R | TGGGCAGAAGGAAATCGACAG |
| MEDIP let7i |  | F | ACTGCCTTGCTAGTGCTGGT |
| MEDIP let7i |  | R | GCGGCTAGCTAACGGTTTC |
| MEDIP miR-219a |  | F | AGACGTCACCGCCCTCAC |
| MEDIP miR-219a |  | R | TTGGGCTCTCCACAAGTACC |
| MEDIP miR-132/212 |  | F | CAAATGCAGACGCAGACACT |
| MEDIP miR-132/212 |  | R | AGCACCTTCTCCCCACTTTC |
| MEDIP miR-207 CPG 1.1 |  | F | GGCTCCGCTACAAAAGAGGT |
| MEDIP miR-207 CPG 1.1 |  | R | AAAGAGGAGCGTAGCACAGC |
| MEDIP miR-207 CPG 1.2 |  | F | GCTGTGCTACGCTCCTCTTT |
| MEDIP miR-207 CPG 1.2 |  | R | GAGGGAGACCCAAGGACCTA |
|  |  |  |  |

|  | **Supplementary Table 5.** miRNAs associated with estrus phase | | | | | | |  |
| --- | --- | --- | --- | --- | --- | --- | --- | --- |
|  |  |  |  | ***CPM-Fold change*** |  |  |  |  |
|  |  | | | | | | |  |
|  |  | Accession ID | miRNA | p/d:e/m | *p* | *Sig.* |  |  |
|  | *estrus phase.* |  |  |  |  |  |  |  |
|  |  | *MIMAT0000800* | *miR-28-5p* | 1.26 | *0.007* | * |  |  |
|  |  | *MIMAT0017357* | *miR-362-3p* | 1.32 | *0.009* | * |  |  |
|  |  | *MIMAT0005309* | *miR-384-5p* | -1.15 | *0.015* | * |  |  |
|  |  | *MIMAT0003208* | *miR-374-5p* | 1.20 | *0.023* | * |  |  |
|  |  | *MIMAT0030409* | *miR-155-5p* | 1.37 | *0.034* | * |  |  |
|  |  | *MIMAT0005282* | *miR-872-5p* | 1.15 | *0.036* | * |  |  |
|  |  |  |  |  |  |  |  |  |

Significantly different miRNAs between estrus phase are listed. Foldchange is shown as a ration between the two estrus phases. **p<0.05; abbreviations: CPM-counts per million, p/d-proestrus/diestrus, e/m-estrus/metestrus.*


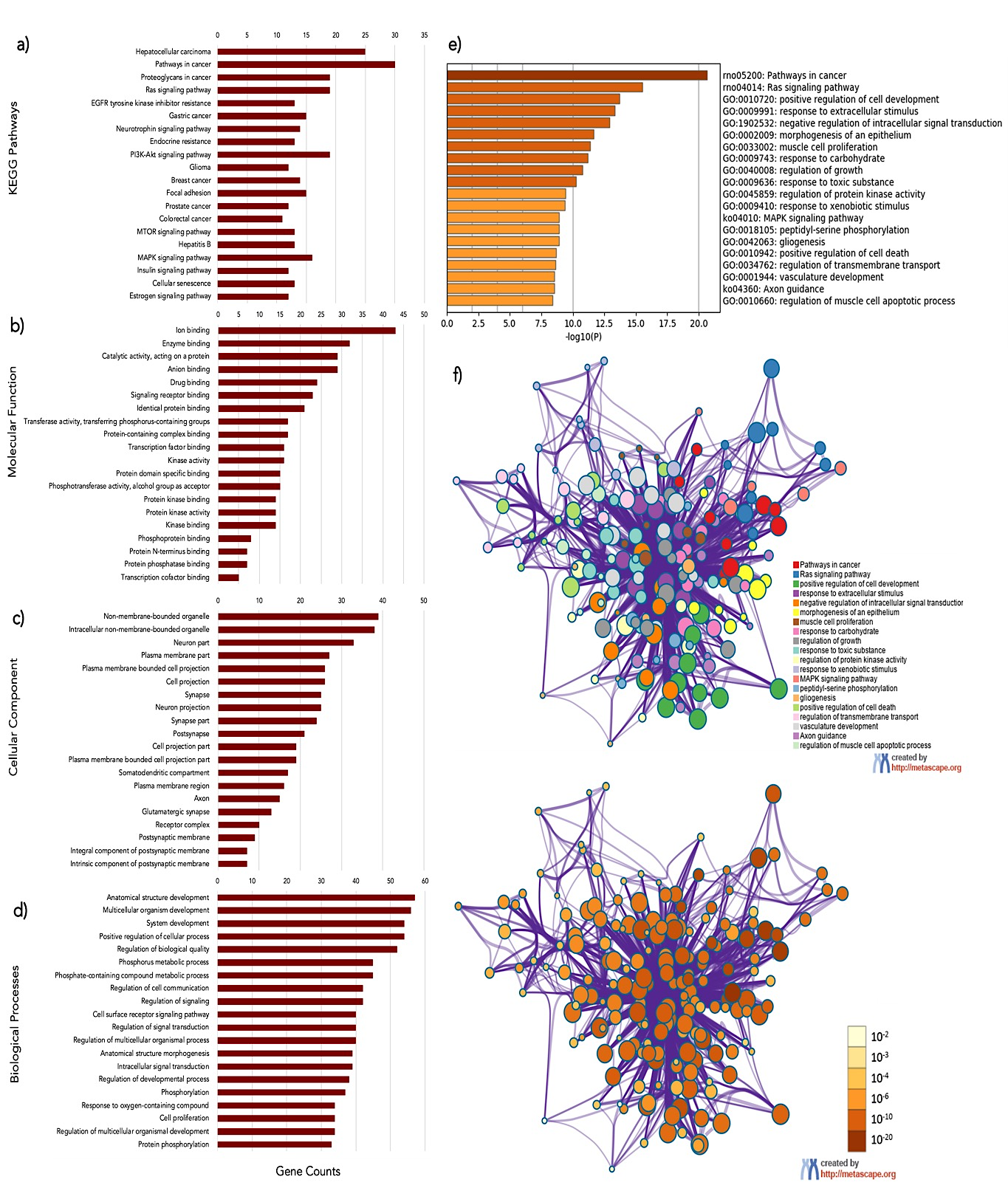
**Supplementary Figure 2. Gene ontologies based on MS miRNA targets.** Gene ontology lists (**a)** KEGG Pathway, **b)** Molecular Function, **c)** Cellular Component, and **d)** Biological Processes were extracted using ShinyGO based on 99 stress-related miRNA gene targets. Ontologies at the top of each list were most significant (*fdr corrected <0.*05) and bars represent the number of genes in each ontology term.
